# Fourier Spot Volatility Estimator: Asymptotic Normality and Efficiency with Liquid and Illiquid High-Frequency Data

**DOI:** 10.1371/journal.pone.0139041

**Published:** 2015-09-30

**Authors:** Maria Elvira Mancino, Maria Cristina Recchioni

**Affiliations:** 1 Department of Economics and Management, University of Florence, Florence, Italy; 2 Department of Management, Polytechnical University of Marche, Ancona, Italy; University College London, UNITED KINGDOM

## Abstract

The recent availability of high frequency data has permitted more efficient ways of computing volatility. However, estimation of volatility from asset price observations is challenging because observed high frequency data are generally affected by noise-microstructure effects. We address this issue by using the Fourier estimator of instantaneous volatility introduced in Malliavin and Mancino 2002. We prove a central limit theorem for this estimator with optimal rate and asymptotic variance. An extensive simulation study shows the accuracy of the spot volatility estimates obtained using the Fourier estimator and its robustness even in the presence of different microstructure noise specifications. An empirical analysis on high frequency data (U.S. *S*&*P*500 and FIB 30 indices) illustrates how the Fourier spot volatility estimates can be successfully used to study intraday variations of volatility and to predict intraday Value at Risk.

## 1 Introduction

The relevance of the estimation of time varying volatility in financial economics has been recognized for a long time but the recent availability of high-frequency financial data has given an enormous impulse to its investigation and application. As a matter of fact, estimates of spot volatility can be used to predict intraday Value at Risk (VaR) and to estimate stochastic volatility model parameters which are necessary for forecasting stock and futures prices, bond yields and so on.

Indeed, estimators capable of replicating satisfactorily intra-day volatility time variations may be useful tools for developing indicators of financial fragility and speculative behaviors. A first step towards this objective is the use of the spot volatility estimates to calibrate agent based models, such as the model proposed in Refs. [1–6]. In fact, knowledge of spot volatility allows us to reconstruct the agents’ fractions giving insights into the main strategies used by the traders in the financial markets considered as illustrated in Refs. [1, 3, 4, 7]. Furthermore, the estimates of spot volatility can also be applied in medical research such as in cardiac and neuronal signal processing. In fact, these estimates could be used to approximate heart rate volatility in order to design an early predictor of malign arrhythmias. In the recent scientific literature, we can find analysis of the short-term (5-minute) heart rate variability (HRV) based on the Fourier and fractal methodology for detecting anomalies in the variations of beat-to-beat interval series [8].

Volatility can be estimated through parametric or nonparametric methods as illustrated in the review [9]. Nonparametric methods address the computation of the historical volatility without assuming a specific functional form of the volatility while trying to reproduce well known stylized facts [7, 10]. Volatility is mainly computed over discrete time intervals relying on the *quadratic variation formula* and thus it is an *integrated volatility*. Instantaneous volatility estimation from high frequency data was first proposed in Ref. [11], by using rolling and block sampling filters. The benchmark for computing the volatility of a financial time series in a time interval with high frequency data (e.g. daily volatility) is provided by the sum of the squared intraday returns (i.e. *realized volatility*) [9, 12, 13]. In the limit, as the time interval between two consecutive observations converges to zero, the realized volatility converges to the quadratic variation of the process and its derivative provides the instantaneous volatility of the process. However, the approximation of the quadratic variation derivative required to get the instantaneous volatility generates appreciable numerical instabilities.

This paper deals with an alternative approach, based on the Fourier series and the Bohr convolution formula. This approach has been introduced in Refs. [14, 15] and it is mainly designed for measuring instantaneous multivariate volatility. The Fourier method reconstructs the instantaneous volatility as a series expansion with coefficients gathered from the Fourier coefficients of the price variation. For this reason it is based on the integration of the time series of returns rather than on its differentiation. Doing so, the Fourier estimator uses all the available observations and avoids any manipulation of the original data and any unstable numerical derivatives.

Several papers have studied the efficiency of the Fourier method in estimating the integrated volatility or co-volatilities even in the presence of microstructure noise, e.g. [16–22]. In fact, by considering the Fourier estimator of 0-th Fourier coefficient of the volatility function we obtain a consistent estimator of the integrated volatility. Moreover, the Fourier methodology has inspired some spot volatility estimators, such as the robust to jumps Fourier estimator in Ref. [23] and the spectral estimator in Ref. [24]. Conversely, the statistical properties and the empirical effectiveness of the Fourier method in estimating the whole path of the volatility process needs to be investigated further. This paper contributes to filling this gap.

Firstly, we prove the pointwise central limit theorem for the spot volatility estimator with a speed of convergence which is the optimal one for a spot volatility estimator. Furthermore, we show how to optimize the asymptotic variance through a suitable choice of ratio of the observation number to the number of the Fourier frequencies. These frequencies are those to be used in the Fejer series to reconstruct the volatility process.

Secondly, we study the efficiency of the Fourier estimator of spot volatility with high frequency data. In particular, we investigate the asymptotic normality of the estimator and the accuracy of the spot volatility estimates using 1-second returns. The accuracy of the estimates is tested comparing the empirical and theoretical distributions of the so called standardized returns [25]. We stress the point that the Fourier estimation method is a *global* method. In fact, it is designed to estimate the volatility path over the entire interval of interest. This fact may be relevant when the estimated volatility is used to calibrate stochastic volatility models. In contrast, most spot volatility estimators, especially the methods based on the quadratic variation formula, are defined as pointwise estimators; thus, their adjustment parameters are tuned to work well only at a specific point in time. Moreover, we show the robustness of the Fourier estimator to various microstructure noise specifications such as the additive noise and the rounding error. We prove that the estimator has a competitive edge even when compared to bias adjusted estimators, such as the Two-Scales realized spot variance estimator [26, 27]. In fact, the Fourier estimator performs very well using high frequency data in all the different scenarios considered and without requiring any *ad hoc* adjustment.

Finally, we conduct an empirical analysis on high frequency liquid and illiquid data. As stressed in Ref. [25], high quality realized variance estimates can be constructed in a liquid market. Nevertheless, we show that the Fourier estimator provides accurate estimates even when illiquid assets are considered. This is probably due to its robustness to rounding errors. In fact, the price of an illiquid asset behaves like a price affected by rounding errors [28]. In addition, we illustrate some exercises to analyze the intraday volatility variations and to predict intraday Value at Risk.

The paper is organized as follows. Section 2 introduces the Fourier estimator. Section 3 contains the central limit theorem. Section 4 illustrates the finite sample properties of the Fourier estimator, including its robustness to microstructure noise. Section 5 presents an empirical analysis conducted on illiquid and liquid data. Section 6 concludes. Finally, Section 7 contains the proof of the central limit theorem and Section 8 summarizes some properties of Fejer and Dirichlet kernels.

## 2 Fourier Estimator of Spot Volatility

In this section we recall the definition of the Fourier estimator of spot volatility introduced in Ref. [14]. However, before to enter the technical definition of the estimator, we introduce the main argument underlying the Fourier methodology, that is the use of the *convolution formula* (3).

Suppose that the asset log-price *p*(*t*) follows a semi-martingale satisfying the Itô stochastic differential equations

$$\begin{array}{c} dp(t)=\sigma (t)\;dW(t)+b(t)\;dt, \end{array}$$ (1)

where *W* is a Brownian motion on a filtered probability space satisfying the *usual conditions*, and *σ* and *b* are adapted random processes such that $E[\int_{0}^{T}b^{2}(t)dt]<\infty$ and $E[\int_{0}^{T}\sigma^{4}(t)dt]<\infty$. Our model is very general, in particular it includes a fairly large class of stochastic volatility models which are widely used in finance, e.g. classical models such as [29–31]. In particular, leverage effects are allowed [32]. We stress the point that our approach is non-parametric, therefore we do not specify any functional form of the volatility process *σ*, we only assume the continuity of the paths (essentially, the Hölder-continuity of any Brownian path).

By change of the origin of time and rescaling the unit of time we can always reduce ourselves to the case where the time window [0, *T*] becomes [0,2*π*].

We now define the Fourier estimator of spot volatility introduced in Ref. [14]. For any positive integer *n*, let 0 = *t*_0_ ≤ ⋯ ≤ *t*_*n*_ = 2*π* be the (possibly unequally-spaced) trading dates of the asset, i.e., the observation times of the asset price. Denote *ρ*(*n*) := max_0 ≤ *i* ≤ *n* − 1_∣*t*_*i*+1_ − *t*_*i*_∣ and suppose that *ρ*(*n*) → 0 as *n* → ∞. Moreover, let *δ*_*i*_(*p*) := *p*(*t*_*i*+1_) − *p*(*t*_*i*_).

For any integer *k*, ∣*k*∣ ≤ 2*N*, define the discrete Fourier transform

$$\begin{array}{c} c_{k}\left(dp_{n}\right):=\frac{1}{2\pi }\sum_{i=0}^{n-1}e^{-ikt_{i}}\delta_{i}\left(p\right), \end{array}$$ (2)

then, for any integer *k*, ∣*k*∣ ≤ *N*, consider the following *convolution* formula

$$\begin{array}{c} c_{k}\left(\sigma_{n,N}^{2}\right):=\frac{2\pi }{2N+1}\sum_{|h|\leq N}c_{h}\left(dp_{n}\right)c_{k-h}\left(dp_{n}\right). \end{array}$$ (3)

(Formula 3) contains the identity relating the Fourier transform of the price process *p*(*t*) to the Fourier transform of the volatility *σ* ^2^(*t*). By Eq (3) we gather all the Fourier coefficients of the volatility function by means of the Fourier transform of the log-returns. Then, the reconstruction of the volatility function *σ* ^2^(*t*) from its Fourier coefficients, can be obtained by the Fourier-Fejer summation. Finally, the *Fourier estimator of spot volatility* is defined: for any *t* ∈ (0,2*π*)

$$\begin{array}{c} {\hat{\sigma }}_{n,N,M}^{2}\left(t\right)=\sum_{|k|\leq M}(1-\frac{\left|k\right|}{M})c_{k}\left(\sigma_{n,N}^{2}\right)\;e^{itk}. \end{array}$$ (4)

We note that the definition of the estimator ${\hat{\sigma }}_{n,N,M}^{2}(t)$ depends on three parameters, the number of data *n* and the two *cutting frequencies* *N*, *M*. The choice of the relative growth of them will be discussed in the following paragraphs.

Note that we can write the estimated Fourier coefficients (3) as

$$\begin{array}{c} c_{k}\left(\sigma_{n,N}^{2}\right)=\frac{1}{2\pi }\sum_{i=0}^{n-1}\sum_{j=0}^{n-1}D_{N}\left(t_{j}-t_{i}\right)e^{-ikt_{j}}\delta_{i}\left(p\right)\delta_{j}\left(p\right), \end{array}$$

where *D*_*N*_ is the rescaled Dirichlet kernel defined as

$$\begin{array}{c} D_{N}\left(x\right)=\frac{1}{2N+1}\sum_{|h|\leq N}e^{ihx}=\frac{1}{2N+1}\frac{\sin\left(2N+1\right)\frac{x}{2}}{\sin\frac{x}{2}}. \end{array}$$ (5)

Thus, the Fourier estimator of spot volatility (4) can be expressed as follows

$$\begin{array}{c} {\hat{\sigma }}_{n,N,M}^{2}\left(t\right)=\frac{1}{2\pi }\sum_{i=0}^{n-1}\sum_{j=0}^{n-1}F_{M}\left(t-t_{j}\right)D_{N}\left(t_{j}-t_{i}\right)\delta_{i}\left(p\right)\delta_{j}\left(p\right), \end{array}$$ (6)

where *F*_*M*_ is the Fejer kernel defined as

$$\begin{array}{c} F_{M}\left(x\right)=\sum_{|k|\leq M}(1-\frac{\left|k\right|}{M})e^{ikx}=\frac{1}{M+1}{\left(\frac{\sin\left(M+1\right)\frac{x}{2}}{\sin\frac{x}{2}}\right)}^{2}. \end{array}$$ (7)

We stress the point that the estimator (6) contains two terms: the quadratic part

$$\begin{array}{c} \frac{1}{2\pi }\sum_{j=0}^{n-1}F_{M}\left(t-t_{j}\right){\left(\delta_{j}\left(p\right)\right)}^{2} \end{array}$$ (8)

and the cross terms

12π∑i=0n-1∑j=0j≠in-1FM(t-tj)DN(tj-ti)δi(p)δj(p). (9)

The quadratic term (8) behaves like the Kernel-based spot volatility estimators seen in Refs. [33, 34]. Nevertheless, the second addend (9) is crucial in terms of robustness of the estimator in the presence of microstructure noise, through the choice of the frequency *N*, as it has also been pointed out for the Realised kernels estimator of integrated variance proposed in Ref. [35] and for the Laplace estimator proposed in Ref. [36]. A comparative analysis of the robustness of the Fourier spot volatility estimator with high frequency data is conducted in Section 4.

**Remark 2.1** *Although we have not considered the Fourier estimator of multivariate volatility in this paper, it is worth noting that the convolution formula* (3) *is directly applicable for obtaining the Fourier estimator of the covariance process between two asset price processes as explained in Ref.* [15]. *Moreover, the advantage of the convolution approach used by the Fourier estimator is that it is immune to the so called *Epps effect** [37]. *In fact, when returns are recorded at the highest available observation frequency, they are asynchronous across different assets* [38]. *The realized covariance type estimators (e.g.* [12, 25, 26, 39]) *require choosing a “synchronization” method. Thus these estimators suffer from a downward bias, when the sampling interval is reduced. On the contrary, the Fourier estimator uses all the available observations and avoids any “synchronization” of the original data, because it is based on the integration of the time series of returns rather than on its differentiation*.

## 3 Asymptotic Normality

In this section we study the pointwise asymptotic error distribution for the Fourier estimator of spot volatility defined in Eq (6). The central limit theorem assumes the ratio $\frac{N}{n}$ between the cutting frequency and the number of data to be asymptotically constant (the Nyquist frequency $N=\frac{n}{2}$ being a reference ratio), while the frequency *M* is slower increasing with respect to *n*. The speed of convergence is the optimal rate of convergence for a spot volatility estimator, at the cost of a (possible) bigger error variance. The discussion on how to optimize the asymptotic variance through a suitable choice of the ratio $\frac{N}{n}$ is contained in Remark 3.3.

The limiting error distribution for the integrated multivariate volatility of the Fourier estimator with asynchronous trading in the absence of microstructure noise is studied in Ref. [21] and in the presence of microstructure noise in Ref. [22]. Note that the Fourier estimation of integrated quantities (integrated volatility or covariance) involves only the 0-th Fourier coefficient in the expansion.

We assume that the volatility process *σ* is a.s. continuous in [0,2*π*] (e.g. driven by a second Brownian semimartingale), more precisely Hölder continuous with parameter $\nu \in (0,\frac{1}{2})$. For simplicity, we consider equally spaced observations, thus $\rho (n)=\frac{2\pi }{n}$.

**Theorem 3.1** *Assume that the following conditions hold*: ${\text{lim}}_{n,M\to \infty }\frac{M^{\gamma }}{n}=a>0$, *for some* *γ* > 1, *and* ${\text{lim}}_{n,N\to \infty }\frac{N}{n}=c>0$. *Then, for any fixed* *t* ∈ (0,2*π*), as *n*, *N*, *M* → ∞,

$$\begin{array}{c} \sqrt{\frac{n}{M}}({\hat{\sigma }}_{n,N,M}^{2}\left(t\right)-\sigma^{2}\left(t\right))\to 𝒩(0,\frac{4}{3}\left(1+2\eta \left(c\right)\right)\;\sigma^{4}\left(t\right)), \end{array}$$

*where the convergence is stable in law and the constant* *η*(*c*) *is defined in* Eq (10).

**Remark 3.2** *(Rate of convergence) The convergence rate in Theorem 3.1 is of order* $n^{\frac{\gamma -1}{2\gamma }}$. *It appears in the proof that* 1 < *γ* < 2*ν* + 1, *where* $\nu \in (0,\frac{1}{2})$. *For* *γ* *close to 2 the rate of convergence becomes* $\frac{1}{4}$, *which is the optimal rate of convergence for a non-parametric spot volatility estimator*.

**Remark 3.3** *(Optimal variance) The constant* *η*(*c*) *is equal to*

$$\begin{array}{c} \eta \left(c\right):=\frac{1}{2{\tilde{c}}^{2}}r\left(\tilde{c}\right)\left(1-r\left(\tilde{c}\right)\right), \end{array}$$ (10)

*where* $\tilde{c}=2c$ *and* *r*(*x*) = *x* − [*x*], *with* [*x*] *the integer part of* *x*. *The computation of the constant* *η*(*c*) *is presented in Lemma 1 formula (26) in Ref*. [21]. *Note that* *η*(*c*) *is nonnegative for any positive* *c* *and equal to zero when* $c=\frac{1}{2}k$, *k* = 1, 2, ….

*The case* *η*(*c*) = 0 *is interesting since it provides the optimal asymptotic variance* $\frac{4}{3}\sigma^{4}(t)$ *as discussed in Ref*. [23]. *The optimal asymptotic variance is obtained for* $c=\frac{1}{2}k$, *k* = 1, 2, … *and the choice* *k* = 1 (*i.e.* $c=\frac{1}{2}$) *corresponds to the natural choice of the Nyquist frequency for the Fourier estimator. Furthermore, for empirical purposes, the number of frequencies used in the Fourier transform is chosen less than the number* *n* *of the available price observations, so that the values* $c=\frac{1}{2}k$, *k* = 2, 3, … *are not effective while the value* $c=\frac{1}{2}$ *is appropriate. We stress that with this choice of* *c* (*in other words of* *N*/*n*) *the Fourier estimator has the same rate of convergence and asymptotic variance of the Fejer kernel-based realized spot volatility considered in Refs*. [34, 40]. *Therefore, with an appropriate choice of* *N*/*n*, *the effect of adding the cross terms in* Eq (6), *which is essential in order to get an estimator robust to microstructure noise, is also not detrimental in view of the asymptotic efficiency. This particular feature of the Fourier estimator is analyzed in Section 4*.

## 4 Simulation studies

In this section we study the efficiency of the Fourier estimator of spot volatility with high frequency data. Firstly, we illustrate some finite sample properties of this estimator. In particular, we investigate the asymptotic normality and the accuracy of the spot volatility estimates using 1-second returns. The accuracy of the estimates is tested by comparing the empirical and theoretical distributions of the so called standardized returns. Secondly, we study the robustness of the Fourier estimator to some microstructure noise specifications, namely, the additive (even dependent) noise and the rounding error. Our analysis shows that it has a competitive edge, even when compared with methods specifically designed to handle market microstructure contaminations.

### 4.1 Finite sample properties

In this section we investigate the finite sample properties of the Fourier spot volatility estimator. Consider the following one factor stochastic volatility model, which is also studied in Ref. [27]:

$$\begin{array}{c} dp(t)=\mu \;dt+\sigma (t)\;dW(t), \end{array}$$ (11)

$$\begin{array}{c} \sigma \left(t\right)=\exp(\beta_{0}+\beta_{1}\;\tau \left(t\right)), \end{array}$$ (12)

$$\begin{array}{c} d\tau \left(t\right)=\beta_{2}\;\tau \left(t\right)\;dt+dZ\left(t\right), \end{array}$$ (13)

where *W* and *Z* are correlated Brownian motions such that 〈*dW*, *dZ*〉_*t*_ = *λ* *dt*. The parameter *β*_0_ is chosen equal to *β*_1_/(2*β*_2_). The initial random variable *τ*_0_ is sampled from the distribution N (0, −1/(2*β*_2_)) and the initial log-price is *p*_0_ = log(9). The values of the parameters are as follows: *μ* = 0.03, *β*_1_ = 0.125, *β*_2_ = −0.025, *λ* = −0.3. The second-by-second return and variance paths over a daily trading period of *T* = 6.5 *hours* = 1 day are computed using the explicit Euler discretization scheme with variable step-size. We simulate 504 trading days with *n* = 23400 observations per day.

**Fig 1:**
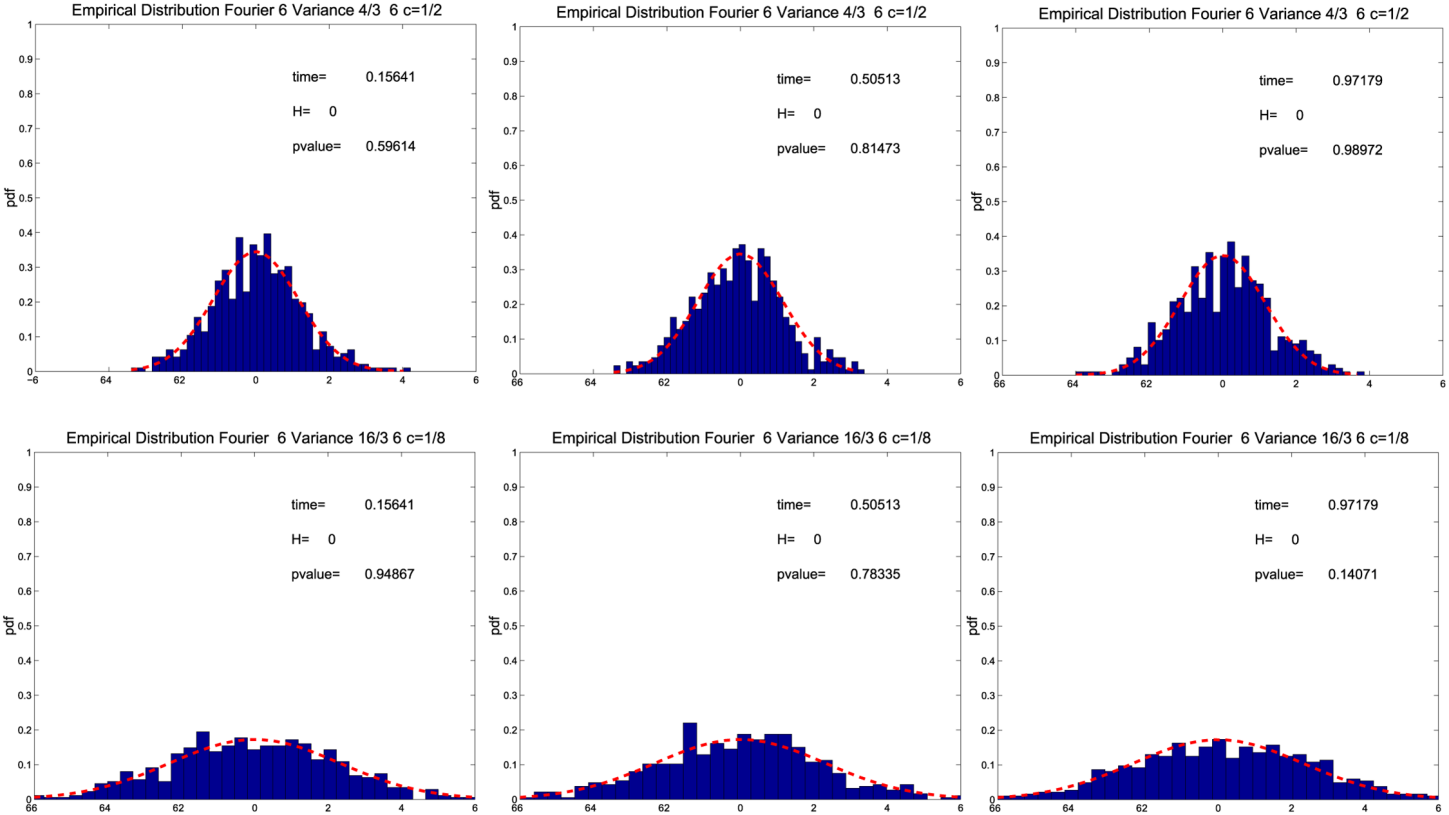
Empirical distribution of the normalized asymptotic error. The panels show the empirical distribution of $\sqrt{n/M}({\hat{\sigma }}^{2}(t)-\sigma^{2}(t))/\sigma^{2}(t)$ for *t* ≈ 0.15 left panels, *t* ≈ 0.5 middle panels, *t* ≈ 0.9 right panels and *c* = 1/2 upper panels, *c* = 1/8 lower panels with 1-second returns.

Firstly, we study the asymptotic error distribution of the Fourier estimator ${\hat{\sigma }}^{2}(t)$. Fig 1 shows the empirical distribution of $\sqrt{n/M}({\hat{\sigma }}^{2}(t)-\sigma^{2}(t))/\sigma^{2}(t)$, when *n* = 23400, *ρ*(*n*) = *T*/*n*, *T* = 1 day, $M=\frac{1}{2\pi }\frac{1}{8}(\sqrt{n}\;\text{log}\;n)$, *N* = *c* *n*. The bandwidth *M* is chosen in order to fulfill the requirement *M* ^*γ*^/*n* = *O*(1) with 1 < *γ* < 2. Our choice is the one used in Ref. [33] to construct realised kernel spot volatility estimators. We consider *c* = 1/2 (i.e. 2*c* = 1) (Fig 1 *t* ≈ 0.15 (left upper panel), *t* ≈ 0.5 (middle upper panel), *t* ≈ 0.9 (right upper panel)) and *c* = 1/8 (i.e. 2*c* = 1/4) (Fig 1 *t* ≈ 0.15 (left lower panel), *t* ≈ 0.5 (middle lower panel), *t* ≈ 0.9 (right lower panel)). In each panel we also show the probability density function $𝓝\left(0,\frac{4}{3}(1+2\eta (c))\right)$ prescribed by Theorem 3.1, where *η*(*c*) is defined in Eq (10). The empirical distributions shown in Fig 1 confirm the findings of Theorem 3.1, namely, the result that the asymptotic distribution of $\sqrt{n/M}({\hat{\sigma }}^{2}(t)-\sigma^{2}(t))/\sigma^{2}(t)$ is $𝓝(0,\frac{4}{3}\;m)$, when *c* = 1/(2*m*), *m* = 1, 2, …. In fact, when *c* = 1/2 the variance attains its smallest value (i.e. 4/3), while the variance is larger when *c* = 1/8. The empirical distributions shown in each panel are tested for normality using the Bera-Jarque test at the significance level of 0.05. The test shows that the null hypothesis is not rejected and the p-values are shown in Fig 1.

**Fig 2:**
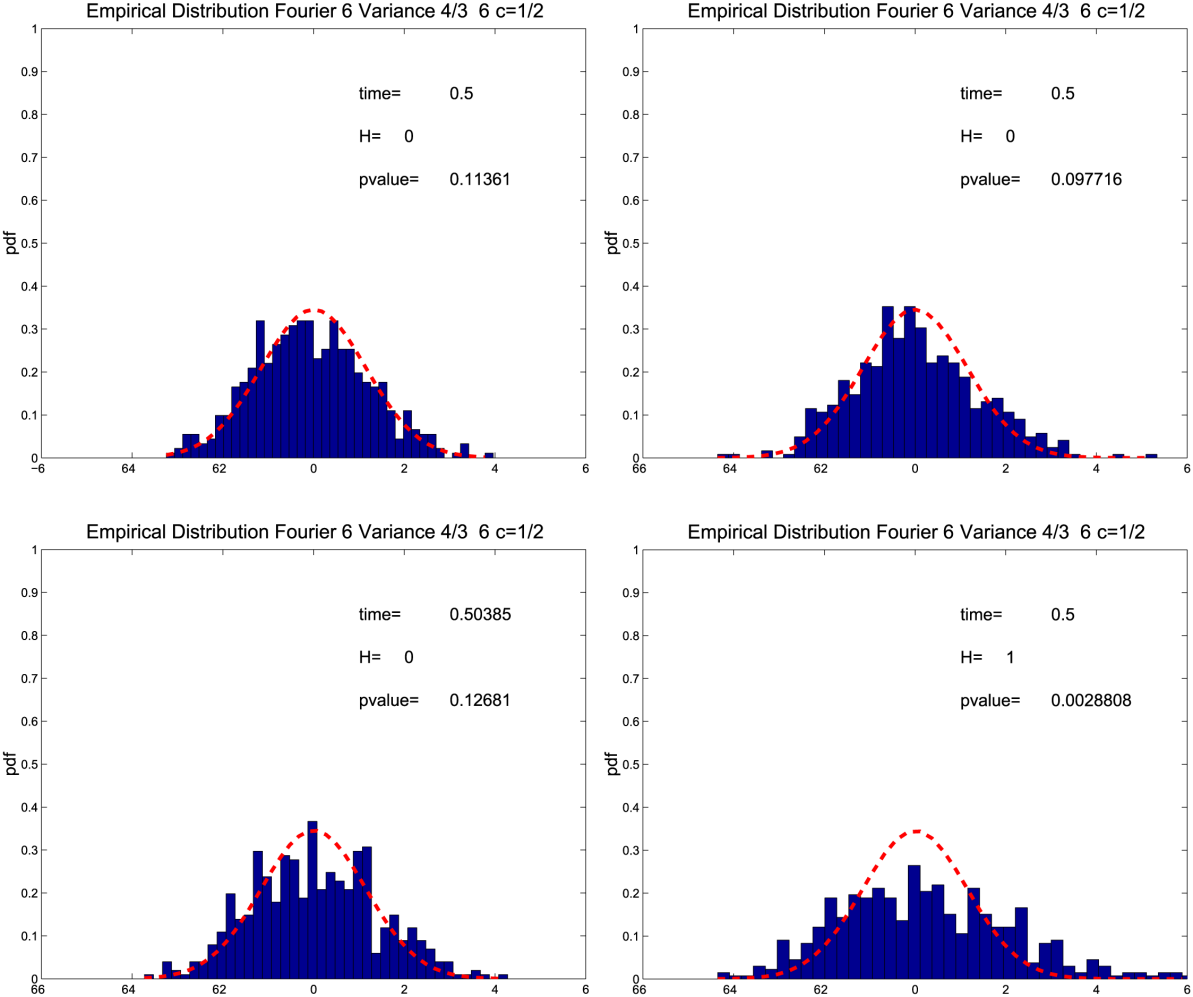
Empirical distribution of the normalized asymptotic error for various sampling intervals. The panels show the empirical distribution of $\sqrt{n/M}({\hat{\sigma }}^{2}(t)-\sigma^{2}(t))/\sigma^{2}(t)$ for *t* ≈ 0.5 and *c* = 1/2 using 1-second returns (left upper panel), 30-second returns (right upper panel), 1-minute returns (left lower panel) 5-minute returns (right lower panel).

We also try to determine the largest price frequency still capable of fitting satisfactorily the theoretical distribution prescribed by Theorem 3.1. We repeat the previous experiment choosing the Nyquist frequency *N* = *n*/2 and $M=\frac{1}{2\pi }\frac{1}{8}(\sqrt{n}\text{log}\;n)$ which are shown to reduce the variance and a sampling interval of ten seconds (i.e. *n* = 23400/10), thirty seconds (i.e. *n* = 23400/30), one minute (i.e. *n* = 23400/60) and five minutes (i.e. *n* = 23400/300). We evaluate the empirical distribution at *t* ≈ 0.5 and we apply the Bera-Jarque test at the significance level of 0.05. The empirical and theoretical distributions are shown in Fig 2. We can observe that when the sampling interval increases from ten seconds to one minute (upper panels and left lower panel) the p-values remain substantially constant while the p-value of the five minute sample deteriorates and the null hypothesis is rejected. This suggests that the finite sample is able to reproduce theoretical properties of $\sqrt{n/M}({\hat{\sigma }}^{2}(t)-\sigma^{2}(t))/\sigma^{2}(t)$ when the price observations are more than one per minute.

**Fig 3:**
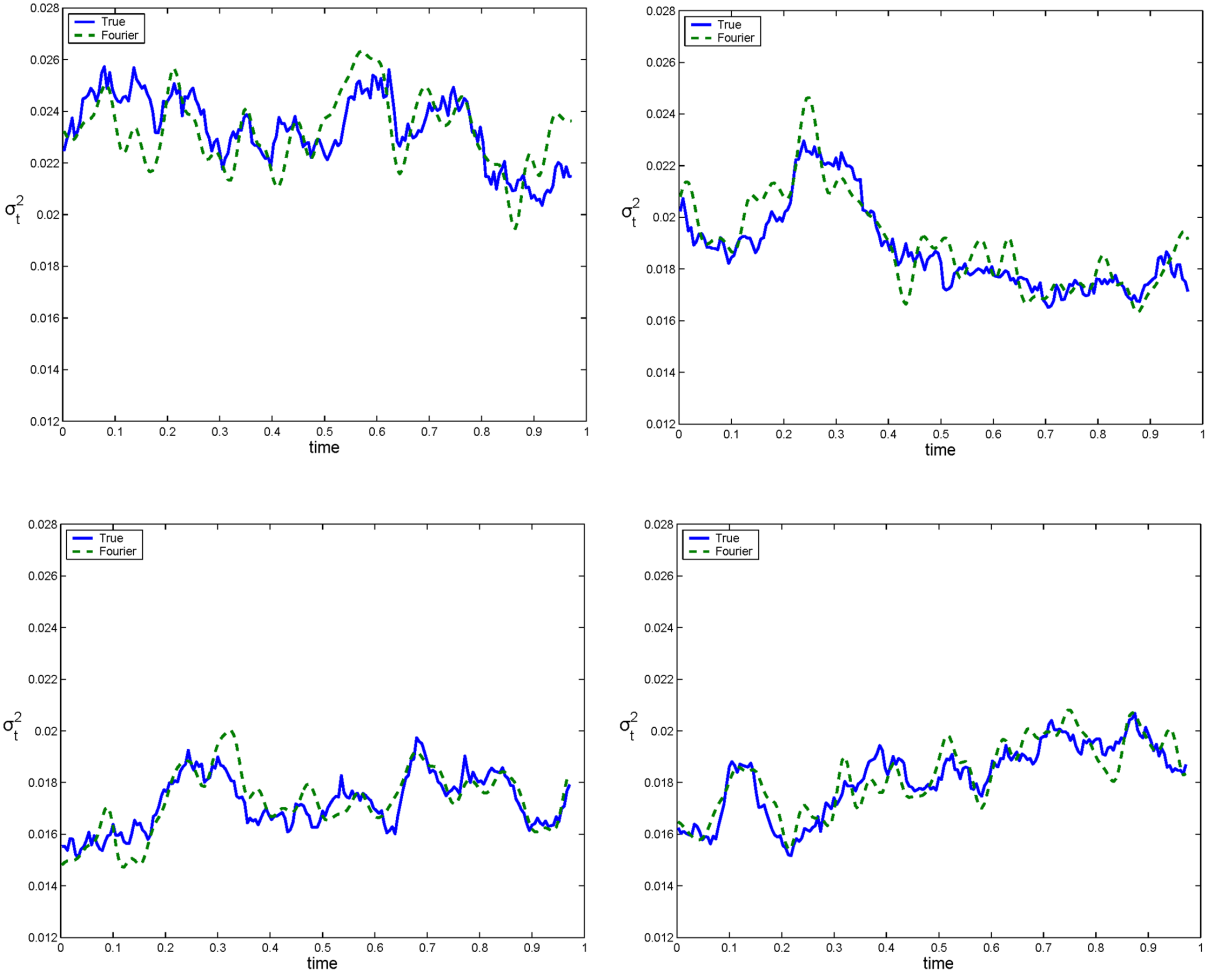
True and estimated variance path. The four graphs show the true variance, *σ* ^2^(*t*), (solid line) and the Fourier estimated variance, ${\hat{\sigma }}^{2}(t)$, (dotted line) as a function of time for four realizations obtained with model (11)–(12) and a sampling interval of 2 minutes.

Secondly, we prove that the Fourier estimator provides accurate spot volatility estimates. We use the simulated data *p*(*t*_*i*_), *t*_*i*_ = *i*/*n*, *i* = 0, 1, …, *n*, *n* = 23400, to estimate the variance *σ* ^2^(*t*) on the time grid $t_{j}^{v}=0.5(2j-1)\Delta \;t$, *j* = 1, 2, …, 23400/120, Δ *t* = 120/23400 = 1/195. That is, we estimate the spot variance using a sampling interval of two minutes. In this exercise the log-prices, *p*(*t*_*i*_) are not affected by microstructure noise. Fig 3 shows four realizations of the true variance (solid line) and the corresponding estimates (dotted line) obtained with the Fourier estimator with *N* = *n*/2 and $M=\frac{1}{2\pi }\frac{1}{8}\sqrt{n}\;\text{log}\;n$. It is worth noting that Fig 3 shows that the Fourier estimator approximates the true variance with a satisfactory accuracy over the entire interval. This property is a consequence of the fact that the Fourier method generates a *global* estimator. This accuracy is relevant for calibrating parametric models such as those of [29–31]. In fact, as shown in the empirical analysis, we can use the spot volatility estimates to reconstruct accurate standardized returns which could be used to efficiently estimate model parameters.

**Fig 4:**
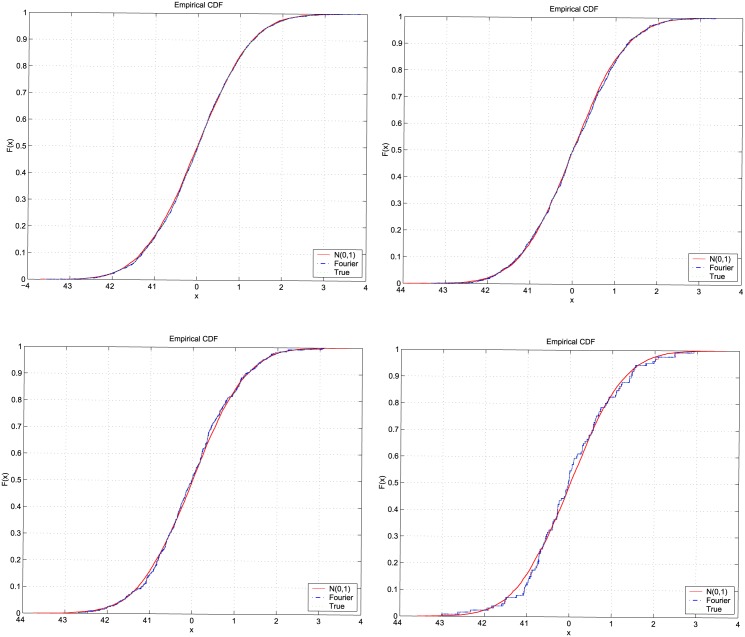
Comparison of cumulative density functions. Cumulative density functions of standard normal sample (red solid line), of the standardized returns obtained using the true volatility (green dotted line) and of the standardized returns obtained using the Fourier spot volatility estimates (blue dash-dot line) when Δ *t* = 10 seconds (left upper panel), Δ *t* = 30 seconds (right upper panel), Δ *t* = 1 minute (left lower panel) and Δ *t* = 3 minutes (right lower panel).

We further investigate the accuracy of the Fourier spot volatility estimates using the standardized returns defined by:

$$\begin{array}{c} z_{t}=\frac{r_{t}}{\sigma_{t}\;\sqrt{\Delta \;t}}\;\;, \end{array}$$ (14)

where *r*_*t*_ = *p*_*t*+Δ *t*_ − *p*_*t*_ is the log-return. These standardized returns are random variables normally distributed with zero mean and variance equal to one when the sample interval is sufficiently small. Specifically, we compute the standardized returns $z_{t}^{\text{true}}$ and $z_{t}^{\text{FR}}$ obtained using the true and the Fourier spot volatilities, respectively, and we compare their cumulative density functions with the theoretical one, N (0,1). Fig 4 shows the results of this comparison when Δ *t* = 10 seconds (Fig 4 left upper panel), Δ *t* = 30 seconds (Fig 4 right upper panel), Δ *t* = 1 minute (Fig 4 left lower panel) and Δ *t* = 3 minutes (Fig 4 right lower panel). The cumulative density functions are obtained elaborating the data of one realization of the log-price variable observed in one day. In addition, we measure the performance of the Fourier estimator by generating 504 replications of the standardized return $z_{t}^{\text{true}}$ and $z_{t}^{\text{FR}}$ for various values of the sampling interval. Then we use the Kolmogorov-Smirnov (KS) and the Jarque-Bera (JB) test at the 5% significance level to determine whether the 504 random samples have the hypothesized standard normal cumulative density function.

Table 1 shows the results of the tests for the sampling intervals Δ *t* = 10 seconds, 30 seconds, 1 minute and 3 minutes. The upper panel in Table 1 shows the sampling interval Δ *t*, the percentage of the KS test rejections, the average p-values, the percentage of the JB test rejections and the average p-values obtained using the true standardized returns $z_{t}^{\text{true}}$, while the lower panel shows the same quantities but for the estimated standardized returns $z_{t}^{\text{FR}}$. We can see that as we increase Δ *t* the percentage of the test rejections increases and the fitting of the cumulative density functions deteriorates (see Fig 4). The poor fit observed when Δ*t* = 3 minutes is due to the fact that the assumption of standardized returns drawn by a standard normal distribution holds only for sufficiently small sampling intervals. When we correct the standardized returns by replacing *r*_*t*_ with *r*_*t*_ − 0.03 Δ *t* in formula (14), the percentage of the test rejections of the KS test and the average p-values are 4% and 0.47 respectively for both samples. For the JB test, the percentage of rejections and the average p-values are 3% and 0.51 respectively for both samples. That is, the percentage of rejections of the two tests is more than halved.

**Table 1:** Comparison of true and estimated standardized returns. We compare the true standardized returns $z_{t}^{\text{true}}$ and the estimated standardized returns $z_{t}^{\text{FR}}$ using Kolmogorov-Smirnov and Jarque-Bera tests.

True standardized returns $z_{t}^{\text{true}}$				
Δ *t*	KS rejections (%)	KS-pvalue	JB rejections (%)	JB-pvalue
10 secs	4%	0.47	6%	0.50
30 secs	3%	0.43	5%	0.48
1 min	8%	0.43	8%	0.48
3 mins	9%	0.46	6%	0.51
Fourier standardized returns $z_{t}^{\text{FR}}$				
Δ *t*	KS rejections (%)	KS-pvalue	JB rejections (%)	JB-pvalue
10 secs	4%	0.47	5%	0.50
30 secs	4%	0.43	5%	0.48
1 min	8%	0.43	8%	0.48
3 mins	10%	0.46	6%	0.51

We highlight that the standardized returns obtained using the true and estimated volatility behave similarly for the four sampling rates considered. This finding indicates that the Fourier estimator is able to reproduce the statistical features of the true spot volatility.

### 4.2 Robustness to microstructure noise effects

In this subsection we illustrate the robustness of the Fourier estimator to various microstructure noise specifications. The simulation study confirms the *global* character of the Fourier estimator. Indeed, it is possible to choose the frequencies *N* and *M* independently of time in order to get accurate spot volatility estimates over the time interval *t* ∈ (0, *T*). Furthermore, even if we choose *M* and *N* independent of the noise specification we still get satisfactory results.

We compare the performance of the Fourier estimator with that of two alternative spot volatility estimators: the Fejer kernel-based realized estimator [33, 34, 40] and the Two-Scales realized spot variance estimator [26, 27]. We choose the Fejer kernel-based realized estimator because it coincides with the *quadratic part* of the Fourier estimator and is given by Eq (8). We show that the Fourier estimator behaves substantially different from the Fejer kernel-based realized estimator, as the presence of the cross term (9) is essential to rendering the estimator efficient in the presence of microstructure noise. The numerical simulations show that the Fourier estimator is robust to microstructure noise effects while the Fejer kernel-based realized estimator is highly biased in the presence of noise. We choose the Two-Scales realized spot variance estimator because it is a suitable competitor to the Fourier estimator when noisy data are considered. In fact, the Two-Scales estimator is constructed by the localization of an integrated volatility estimator specifically designed to be robust to microstructure noise [26].

For the reader’s convenience we recall the definition of these two estimators. The Fejer kernel-based realized estimator is the quadratic part of the Fourier estimator given in Eq (8). We will denote it as ${\hat{\sigma }}_{KF}^{2}(t)$. The Two-Scales realized spot variance estimator (TS hereafter) has the following form:

$$\begin{array}{c} {\hat{\sigma }}_{TS}^{2}\left(t\right):=\frac{1}{h}\sum_{t-h\leq t_{i}\leq t}\frac{{\left(p\left(t_{i}\right)-p\left(t_{i-R}\right)\right)}^{2}}{R}-(\frac{nh-R+1}{nRh})\frac{1}{h}\sum_{t-h\leq t_{i}\leq t}{\left(p\left(t_{i}\right)-p\left(t_{i-1}\right)\right)}^{2}. \end{array}$$ (15)

The parameter *R* is the sub-sampling size parameter (*scale parameter*) and *h* is the interval length parameter (*bandwidth parameter*) [27]. The parameters *R* and *h* play a relevant role as the parameters *N* and 1/*M*, which appear in the Fourier estimator’s definition. We rescale the Fourier and Fejer kernel-based realized estimators by the quantity $\sum_{i=1}^{n}F_{M}(t-t_{i})(t_{i}-t_{i-1})$, where *F*_*M*_ is the Fejer kernel. This scaling improves the performance of both estimators without changing their asymptotic properties [34, 40].

Let us now describe the data-set considered in this subsection. The log-prices, *p*(*t*_*i*_), *i* = 0, 1, …, *n*, are generated simulating the following stochastic volatility model:

$$\begin{array}{c} dp\left(t\right)=\left(\mu -\sigma {\left(t\right)}^{2}/2\right)dt+\sigma \left(t\right)\;dW\left(t\right), \end{array}$$ (16)

$$\begin{array}{c} d\sigma^{2}\left(t\right)=\gamma \;\left(\theta -\sigma^{2}\left(t\right)\right)dt+\nu \sigma \left(t\right)\;dZ\left(t\right), \end{array}$$ (17)

where *W*(*t*) and *Z*(*t*) are standard Brownian motions with correlation *λ* (i.e. 〈*dW*, *dZ*〉_*t*_ = *λ* *dt*). This model (with jumps) has been simulated in Ref. [28] to study the effect of rounding errors on integrated volatility estimators. Following [28], we choose *T* = 1, *ν* = 0.5/252, *γ* = 5/252, *θ* = 0.1, *μ* = 0.05/252, *λ* = −0.5, $\sigma_{0}^{2}=1$, *p*(0) = log(9).

We now introduce the four models of microstructure noise considered in the simulation study. The first three kinds consist of additive noises experienced in the financial prices and already extensively analyzed in the literature ([35, 41–43] and the reference therein). The last one is the rounding error [28, 44]. The rounding error is very critical when high frequency data are used. In fact, the financial prices are often rounded at 1% and this can result in a “piecewise constant” time series. In the first three specifications we suppose that the logarithm of the observed price $\tilde{p}(t_{i})$ is given by:

$$\begin{array}{c} \tilde{p}\left(t_{i}\right)=p\left(t_{i}\right)+\eta \left(t_{i}\right),\;\;\;i=0,\ldots ,n, \end{array}$$ (18)

where *p* is the efficient (latent) log-price process defined by Eq (16) and *η* describes the microstructure noise component.

The first noise specification, (M), is based on the following assumptions:

(M.I) the random shocks *η*(*t*_*i*_) for any *i* = 0, 1, …, *n* are independent and identically distributed with Gaussian distribution $𝓝(0,{\tilde{\eta }}^{2})$
(M.II) the true return process *δ*_*i*_(*p*) is independent of *η*(*t*_*i*_) for any *i* = 0, 1, …, *n* − 1 and for any *n*.

The second specification, (M)′, assumes (M.I)′ and (M.II), where

(M.I)′ the random shocks *η*(*t*_*i*_) for any *i* = 0, 1, …, *n* are allowed for negative first order autocorrelation of the random shocks.

The third noise specification, (MD), is based on (M.I) and (M.II)′, where

(M.II)′ the random shocks *η*(*t*_*i*_) are linearly dependent on the return *δ*_*i*_(*p*), namely:
  $$\begin{array}{c} \eta \left(t_{i}\right)=\zeta \delta_{i}\left(p\right)+{\hat{\eta }}_{i}\;, \end{array}$$
  where ${\hat{\eta }}_{i}$ are Gaussian i.i.d. random variables with variance equal to ${\tilde{\eta }}^{2}$ for any *i* and *ζ* = 0.1. When we consider these three noise specifications, we choose $\tilde{\eta }=\xi \;std(r)$ where *std*(*r*) is the standard deviation of the 1-second returns. The quantity *ξ* is the so called noise-to-signal ratio (see Ref. [42] for further details).

The last noise specification, (MR), takes into account the fact that prices involve rounding errors. The observed log-price are defined as follows:

$$\begin{array}{c} \tilde{p}\left(t_{i}\right)=\log([\frac{\exp(p\left(t_{i}\right))}{l_{\alpha }}]l_{\alpha }),\;\;\;i=0,\ldots ,n, \end{array}$$ (19)

where [⋅] denotes rounding to the nearest integer and *l*_*α*_ is the fixed rounding error level. As highlighted in Ref. [28], given that stock prices are often rounded to the cent, the choice *l*_*α*_ = 0.01 mimics the financial markets.

We apply the explicit Euler discretization scheme with variable step-size to compute second-by-second return and variance paths over a daily trading period of *T* = 1 day. We simulate a total of 504 trading days (about two years) and *n* = 23400 observations per day (approximately one observation per second). The volatility is estimated using a sampling interval of 1 minute since this sampling has shown a good accuracy of the standardized returns (see Fig 4 in Section 4.1).

We measure the performance of the spot volatility estimator, ${\hat{\sigma }}^{2}(t)$, over the entire interval [0, *T*] and near the right boundary by evaluating numerically the relative mean squared error $\text{RMSE}(t)=E\left[{\left({\hat{\sigma }}^{2}(t)-\sigma^{2}(t)\right)}^{2}/\sigma^{4}(t)\right]$ and the bias $\text{BIAS}(t)=E\left[{\hat{\sigma }}^{2}(t)-\sigma^{2}(t)\right]$. Specifically, the performance over the interval [0, *T*] is evaluated using the integrated relative mean squared error

$$\text{IRMSE}=\frac{1}{T}\int_{0}^{T}E[{\left({\hat{\sigma }}^{2}\left(t\right)-\sigma^{2}\left(t\right)\right)}^{2}/\sigma^{4}\left(t\right)]dt$$

and the integrated bias

$$\text{IBIAS}=\frac{1}{T}\int_{0}^{T}E[{\hat{\sigma }}^{2}\left(t\right)-\sigma^{2}\left(t\right)]dt\;.$$

We investigate whether there exists an easily implementable formula for choosing the so called *cutting frequencies* *N* and *M* of the Fourier estimator and the bandwidths *R* and *h* of the TS estimator in order to minimize the integrated relative mean squared error and the relative error at *t* = *T*_*b*_ = 0.98. More specifically, we explore how the optimal choice of *N*, *M*, *R* and *h* depends on *n*. Consider the following values of *N* and *M*:

$$\begin{array}{c} N=\;c_{N}\;n^{\alpha },\;\alpha =1,\;\frac{2}{3},\;\frac{1}{2},\;\frac{1}{3},\;\frac{1}{4}, \end{array}$$ (20)

$$\begin{array}{c} M=c_{M}\;n^{\beta },\;\beta =\frac{2}{3},\;\frac{1}{2},\;\frac{1}{3},\;\frac{1}{4},\;\frac{1}{6},\;\frac{1}{8}, \end{array}$$ (21)

where *c*_*N*_ and *c*_*M*_ are positive constants. We choose $c_{N}=\frac{1}{2}$ and $c_{M}=\frac{1}{2\pi }\frac{1}{8}$ for the Fourier estimator and $c_{M}=\frac{1}{2\pi }\;\frac{1}{8}$ for the Fejer kernel-based realized estimator which depends only on the parameter *M*. We note that *β* := 1/*γ*, where *γ* is the parameter used in Theorem 3.1 to parameterize the cutting frequency *M*. Theorem 3.1 requires $\frac{1}{2}<\beta <1$. In our exercise we also explore the behavior of the Fourier estimator for values of *β* not within the interval prescribed by the central limit theorem. This is theoretically justified by the fact that the highest frequencies should be cut in order to filter out microstructure noise effects arising from high frequency data as shown in Ref. [20]. We check whether 1-second observed data are able to match the asymptotic behavior.

The parameters *R* and *h* of the TS estimator are chosen in a slightly different way by taking into consideration the plug-in approach illustrated in Ref. [27]. More precisely, we choose the following values of *R* and *h*:

$$\begin{array}{c} R=\;c_{R}\;n^{\alpha },\;\;\;\alpha =\frac{2}{3},\;\frac{1}{2},\;\frac{1}{3},\;\frac{1}{4},\;c_{R}=0.05\left(\xi +1\right)/i,\;i=1,2,\ldots ,10, \end{array}$$ (22)

$$\begin{array}{c} h=c_{h}/n^{\beta },\;\;\;\beta =\frac{2}{3},\;\frac{1}{2},\;\frac{1}{3},\;\frac{1}{4},\;\frac{1}{6},\;\frac{1}{8},\;c_{h}=0.5\;i/\left(\xi +1\right),\;\;i=1,2,\ldots ,10, \end{array}$$ (23)

where *ξ* is the noise to signal ratio. Note that, for a fixed value of *n*, we consider forty values of *R* and sixty values of *h*. This is done since we want to explore not only the dependence of *R* and *h* on *n* but also on the noise level *ξ*. For this reason we allow the constants *c*_*R*_ and *c*_*h*_ to vary with the noise level. Tuning the constants *c*_*R*_ and *c*_*h*_ on the noise level strongly improves the performance of the TS estimator, while this is not needed in the case of the Fourier estimator, which turns out to have a good performance even without such *ad hoc* choices of the constants *c*_*N*_ and *c*_*M*_.

Note that we are looking for a bandwidth/frequency choice that can provide satisfactory volatility approximations over the entire time interval without being dependent on time and on specific properties of the volatility process. This fact may be relevant when the estimated volatility is used to calibrate stochastic volatility models. In fact, given the sample, if the value of *M* and *N* are independent of time (and, possibly, of noise) the estimator ${\hat{\sigma }}^{2}(t)$ is a continuous function of *t* and this makes it suitable for the calibration of stochastic volatility models.

We examine the performance of the Fourier estimator, the Fejer-kernel based estimator and the TS estimator both in the absence (*ξ* = 0) and in the presence of noise. In the simulations conducted by using the first three noise specifications, we consider two values of the noise-to-signal ratio, *ξ* = 0.8 and *ξ* = 3.2. That is, we consider a total of six different noises for the additive case. Regarding the fourth specification (i.e. rounded prices) we consider only the rounding levels *l*_*α*_ = 0.01 (commonly observable in financial prices) and *l*_*α*_ = 0.1. Tables 2, 3 and 4 show, from left to right, the noise to signal ratio, the pair (*α* ^*i*^, *β* ^*i*^) which minimizes the integrated mean squared error, the integrated bias, the pair (*α* ^*b*^, *β* ^*b*^) which minimizes the mean square error and the bias at *t* = *T*_*b*_.

**Table 2:** Robustness of the Fourier estimator under microstructure noise effects. The sampling interval is 1 second.

Fourier estimator						
No Noise						
Noise-to-signal ratio *ξ*	(*α* ^*i*^, *β* ^*i*^)	IRMSE	IBIAS	(*α* ^*b*^, *β* ^*b*^)	RMSE(*T*_*b*_)	BIAS(*T*_*b*_)
0.0 (no noise)	$\left(1,\frac{2}{3}\right)$	3.08e-4	-8.64e-4	$\left(1,\frac{2}{3}\right)$	3.32e-4	-7.31e-3
Noise specification (M.I)-(M.II)						
Noise-to-signal ratio *ξ*	(*α* ^*i*^, *β* ^*i*^)	IRMSE	IBIAS	(*α* ^*b*^, *β* ^*b*^)	RMSE(*T*_*b*_)	BIAS(*T*_*b*_)
0.8	$\left(\frac{2}{3},\frac{2}{3}\right)$	8.58e-3	4.38e-3	$\left(\frac{2}{3},\frac{2}{3}\right)$	8.64e-3	1.87e-2
3.2	$\left(\frac{2}{3},\frac{2}{3}\right)$	2.06e-2	4.86e-2	$\left(\frac{2}{3},\frac{2}{3}\right)$	1.84e-2	6.55e-2
Noise specification (M.I)′-(M.II)						
Noise-to-signal ratio *ξ*	(*α* ^*i*^, *β* ^*i*^)	IRMSE	IBIAS	(*α* ^*b*^, *β* ^*b*^)	RMSE(*T*_*b*_)	BIAS(*T*_*b*_)
0.8	$\left(\frac{2}{3},\frac{2}{3}\right)$	8.48e-3	2.22e-3	$\left(\frac{2}{3},\frac{2}{3}\right)$	8.43e-3	1.64e-2
3.2	$\left(\frac{2}{3},\frac{2}{3}\right)$	1.83e-2	1.67e-2	$\left(\frac{2}{3},\frac{2}{3}\right)$	1.38e-2	3.38e-2
Noise specification (M.I)-(M.II)′						
Noise-to-signal ratio *ξ*	(*α* ^*i*^, *β* ^*i*^)	IRMSE	IBIAS	(*α* ^*b*^, *β* ^*b*^)	RMSE(*T*_*b*_)	BIAS(*T*_*b*_)
0.8	$\left(\frac{2}{3},\frac{2}{3}\right)$	8.59e-3	5.01e-3	$\left(\frac{2}{3},\frac{2}{3}\right)$	8.61e-3	1.95e-2
3.2	$\left(\frac{2}{3},\frac{2}{3}\right)$	1.98e-2	4.98e-2	$\left(\frac{2}{3},\frac{2}{3}\right)$	1.73e-2	6.81e-2
Noise specification (MR)						
Rounding level *l*_*α*_	(*α* ^*i*^, *β* ^*i*^)	IRMSE	IBIAS	(*α* ^*b*^, *β* ^*b*^)	RMSE(*T*_*b*_)	BIAS(*T*_*b*_)
0.01	$\left(\frac{2}{3},\frac{2}{3}\right)$	8.36e-3	3.15e-3	$\left(\frac{2}{3},\frac{2}{3}\right)$	8.51e-3	1.75e-2
0.1	$\left(\frac{1}{2},\frac{1}{2}\right)$	4.99e-2	3.36e-2	$\left(\frac{1}{2},\frac{1}{2}\right)$	1.55e-2	4.25e-2

**Table 3:** Robustness of the Fejer-kernel based realized estimator to microstructure noise effects. The sampling interval is 1 second.

Fejer-kernel based realized estimator						
No Noise						
Noise-to-signal ratio *ξ*	*β* ^*i*^	IRMSE	IBIAS	*β* ^*b*^	RMSE(*T*_*b*_)	BIAS(*T*_*b*_)
0.0 (no noise)	$\frac{1}{2}$	2.19e-4	-1.01e-3	$\frac{1}{2}$	2.64e-4	7.49e-3
Noise specification (M.I)-(M.II)						
Noise-to-signal ratio *ξ*	*β* ^*i*^	IRMSE	IBIAS	*β* ^*b*^	RMSE(*T*_*b*_)	BIAS(*T*_*b*_)
0.8	$\frac{1}{6}$	1.01e+1	3.16e+0	$\frac{1}{6}$	1.01e+1	3.16e+0
3.2	$\frac{1}{6}$	2.29e+3	4.73e+1	$\frac{2}{3}$	2.23e+3	4.72e+1
Noise specification (M.I)′-(M.II)						
Noise-to-signal ratio *ξ*	*β* ^*i*^	IRMSE	IBIAS	*β* ^*b*^	RMSE(*T*_*b*_)	BIAS(*T*_*b*_)
0.8	$\frac{1}{6}$	4.90e+0	2.21e+0	$\frac{1}{2}$	4.89e+0	2.12e+0
3.2	$\frac{1}{6}$	1.25e+3	3.54e+1	$\frac{1}{2}$	1.25e+3	3.53e+1
Noise specification (M.I)-(M.II)′						
Noise-to-signal ratio *ξ*	*β* ^*i*^	IRMSE	IBIAS	*β* ^*b*^	RMSE(*T*_*b*_)	BIAS(*T*_*b*_)
0.8	$\frac{1}{3}$	2.19e+0	1.48e+0	$\frac{1}{3}$	2.19e+0	1.48e+0
3.2	$\frac{1}{6}$	4.18e+3	2.04e+1	$\frac{1}{2}$	4.18e+3	2.04e+1
Noise specification (MR)						
Rounding level *l*_*α*_	*β* ^*i*^	IRMSE	IBIAS	*β* ^*b*^	RMSE(*T*_*b*_)	BIAS(*T*_*b*_)
0.01	$\frac{1}{2}$	9.59e-2	2.58e-1	$\frac{1}{2}$	2.27e-1	3.15e-1
0.1	$\frac{1}{2}$	1.58e+1	2.17e+0	$\frac{1}{2}$	4.75e+1	3.57e+0

**Table 4:** Robustness of the Two-Scales realized spot variance estimator to microstructure noise effects. The sampling interval is 1 second.

Two Scales Realized Spot variance estimator						
No Noise						
Noise-to-signal ratio *ξ*	(*α* ^*i*^, *β* ^*i*^)	IRMSE	IBIAS	(*α* ^*b*^, *β* ^*b*^)	RMSE(*T*_*b*_)	BIAS(*T*_*b*_)
0.0 (no noise)	$\left(\frac{2}{3},\frac{1}{6}\right)$	4.73e-2	-9.60e-2	$\left(\frac{2}{3},\frac{1}{6}\right)$	3.92e-3	-4.50e-2
Noise specification (M.I)-(M.II)						
Noise-to-signal ratio *ξ*	(*α* ^*i*^, *β* ^*i*^)	IRMSE	IBIAS	(*α* ^*b*^, *β* ^*b*^)	RMSE(*T*_*b*_)	BIAS(*T*_*b*_)
0.8	$\left(\frac{2}{3},\frac{1}{6}\right)$	3.87e-2	-9.77e-2	$\left(\frac{2}{3},\frac{1}{6}\right)$	1.31e-2	-6.48e-2
3.2	$\left(\frac{2}{3},\frac{1}{6}\right)$	5.46e-2	-6.54e-2	$\left(\frac{2}{3},\frac{1}{6}\right)$	1.41e-2	-1.71e-2
Noise specification (M.I)′-(M.II)						
Noise-to-signal ratio *ξ*	(*α* ^*i*^, *β* ^*i*^)	IRMSE	IBIAS	(*α* ^*b*^, *β* ^*b*^)	RMSE(*T*_*b*_)	BIAS(*T*_*b*_)
0.8	$\left(\frac{2}{3},\frac{1}{6}\right)$	4.66e-2	-1.16e-1	$\left(\frac{2}{3},\frac{1}{6}\right)$	7.86e-3	-3.79e-2
3.2	$\left(\frac{2}{3},\frac{1}{6}\right)$	1.14e-1	-1.73e-1	$\left(\frac{2}{3},\frac{1}{6}\right)$	4.39e-2	-1.19e-1
Noise specification (M.I)-(M.II)′						
Noise-to-signal ratio *ξ*	(*α* ^*i*^, *β* ^*i*^)	IRMSE	IBIAS	(*α* ^*b*^, *β* ^*b*^)	RMSE(*T*_*b*_)	BIAS(*T*_*b*_)
0.8	$\left(\frac{2}{3},\frac{1}{6}\right)$	3.89e-2	-9.85e-2	$\left(\frac{2}{3},\frac{1}{6}\right)$	5.35e-3	-3.48e-2
3.2	$\left(\frac{2}{3},\frac{1}{6}\right)$	5.47e-2	-6.53e-2	$\left(\frac{2}{3},\frac{1}{6}\right)$	1.41e-2	-1.70e-2
Noise specification (MR)						
Rounding level *l*_*α*_	(*α* ^*i*^, *β* ^*i*^)	IRMSE	IBIAS	(*α* ^*b*^, *β* ^*b*^)	RMSE(*T*_*b*_)	BIAS(*T*_*b*_)
0.01	$\left(\frac{2}{3},\frac{1}{6}\right)$	4.13e-2	-1.06e-1	$\left(\frac{2}{3},\frac{1}{6}\right)$	5.29e-3	-3.26e-2
0.1	$\left(\frac{2}{3},\frac{1}{6}\right)$	1.02e-1	-5.67e-2	$\left(\frac{2}{3},\frac{1}{6}\right)$	6.61e-2	6.95e-2

We can see that both the Fourier and the TS estimators are very robust to any noise specifications considered. The TS estimator performs slightly worse than the Fourier one in approximating the volatility over the entire interval, while the TS volatility estimates near the boundary are slightly more accurate than the Fourier ones. This is probably due to the fact that the bandwidths, *R* and *h*, are chosen independently of some specific properties of the volatility process (e.g., integrated quarticity, integrated volatility of volatility, as in Ref. [27]). In other words, the Fourier estimator fits the term *global* slightly better than the TS estimator. Furthermore, the comparison between the results shown in Tables 2 and 3 confirms that the absence of the cross terms appearing in the Fourier estimator significantly affects its robustness.

The test used to select the optimal pair (*α*, *β*) is infeasible since it is based on the minimization of the unknown mean squared error. However, in view of the empirical applications, we propose an elementary feasible test for the bandwidth (frequency) selection. For each *t* and for each pair (*α*, *β*) we construct 504 realization of the standardized log-return $z_{t}^{\alpha ,\beta }$ and we apply the Bera-Jarque test at the significance level 0.05 with null hypothesis that $z_{t}^{\alpha ,\beta }$ is normal with unspecified mean and variance. We compute the rejection percentage of the null hypothesis for each pair (*α*, *β*) and we select the optimal pair to be the one with the smallest rejection percentage. Proceeding with this simple test the selected optimal pairs of the Fourier estimator are the same as those shown in Table 2. Indeed, there is one exception at the rounding error at level 0.1 where the optimal pair selected by the new test is (2/3,1/4). The smallest percentage of rejection varies in the interval [5%, 85%] depending on the noise specifications.

## 5 Empirical Analysis

In this section we present an empirical analysis conducted on illiquid and liquid data to illustrate the ability of the Fourier spot volatility estimator to capture intraday variations of volatility and to predict high-frequency Value-at-Risk (VaR) [26–28].

The same empirical analysis is carried on two data-sets and we have *T* = 1 day. The first data-set consists of 5-second observations of the U.S. S&P 500 index obtained by Bloomberg. We consider 5 trading days from March 4, 2013 to March 7, 2013 corresponding to about one week data. Each trading day starts at 15.30 (Rome local time) and ends at 22:30 (Rome local time). We use the observations from 15:30 to 22:00 since the others remain substantially unchanged. This choice corresponds to 4680 (5-second) observations. The second data-set consists of the nearby Italian stock index futures, FIB30 in January 2001 observed every 5.67 seconds. Specifically, we consider four consecutive trading days, January 27, 28, 29 and 30, 2001. As shown in subsection 5.1 the first data-set is characterized by illiquid data while the second one by liquid data.

We will show that the Fourier estimator has a good performance when both illiquid and liquid data are used. Furthermore, the optimal frequencies *M* and *N* are chosen by using the simple test illustrated in Section 4.2.

### 5.1 Intraday variations of volatility when data are illiquid

**Fig 5:**
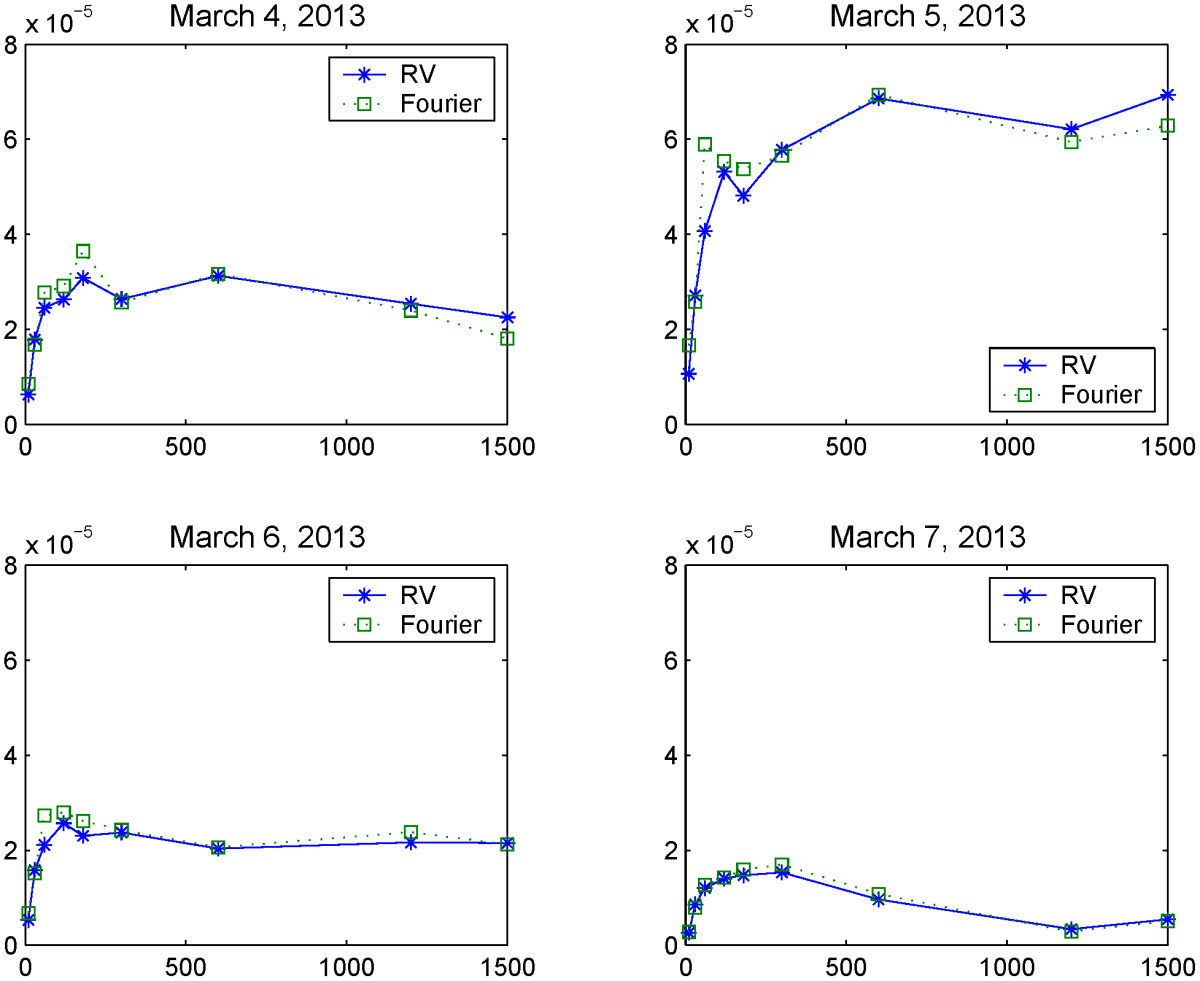
Volatility signature plots versus sampling frequency in seconds. The graphs show the signature plots of illiquid market data on March 4-7, 2013.

In this subsection we consider the first data-set of U.S. S&P 500 index 5-second returns observed on March 4-7, 2013. We first carry out a preliminary analysis to study the main features of the observed data. This analysis is conducted through the volatility signature plot and the autocorrelation functions. Fig 5 shows the volatility signature plots corresponding to March 4, 5, 6 and 7, 2013 evaluated using the Realized Volatility (RV) and the Fourier estimator for integrated volatility. Roughly speaking, the volatility signature plots of Fig 5 are plots of the realized variance against sampling intervals and, as explained in Ref. [25], these sampling intervals are chosen to be multiples of the smallest sampling interval. Ref. [25] highlights that highly liquid assets display the largest realized variance estimates at the highest sampling rates (i.e. 5-second returns) while illiquid assets display the largest realized variance estimates at the lowest sampling rates (i.e. 20-minute returns). In fact, liquid assets show a negative serial autocorrelation so that the oscillating swings in the returns reduce for larger sampling intervals by the effect of cancelation. The signature plots illustrated in Fig 5 show that we are dealing with an illiquid asset.

**Fig 6:**
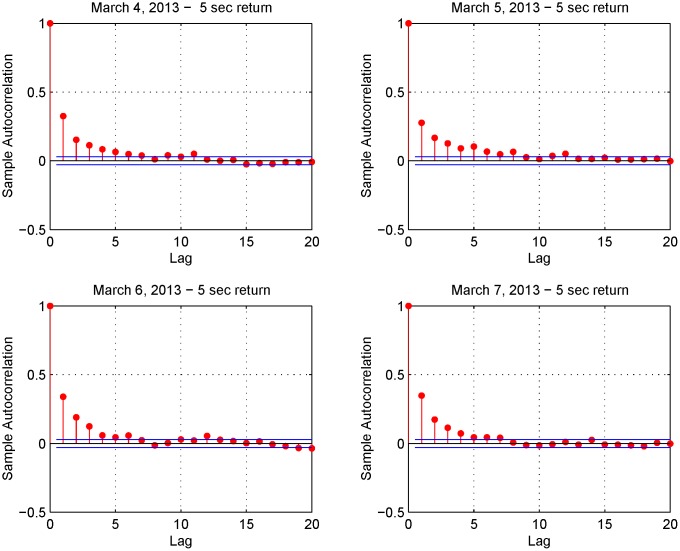
Autocorrelation functions. The four panels show the autocorrelation in 5-second returns (March 4-7, 2013).

**Fig 7:**
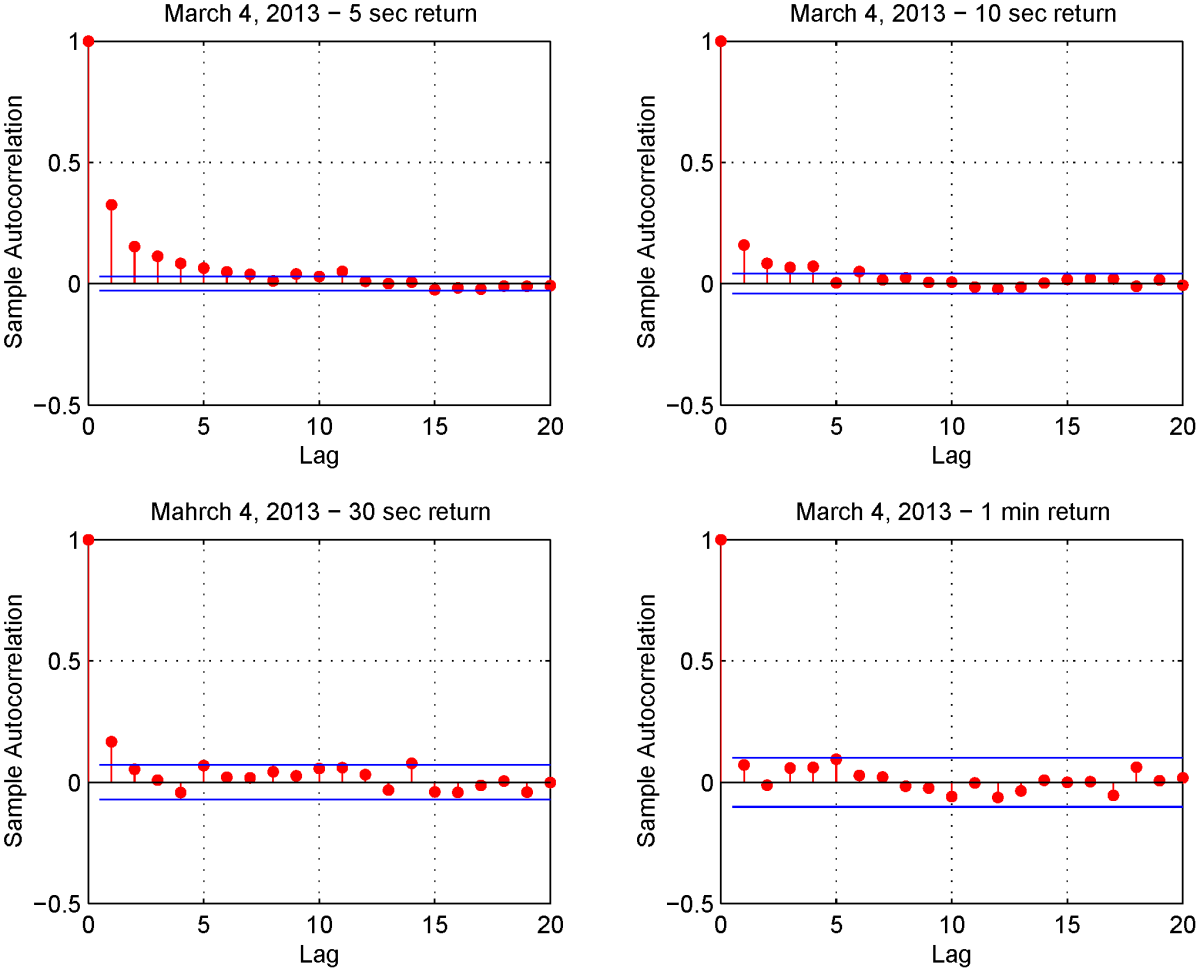
Autocorrelation for various sampling intervals on March 4, 2013. Upper left panel (5-second returns), upper right panel (10-second returns), lower left panel (30-second returns) and lower right panel (1-minute returns).

This finding is confirmed by Figs 6 and 7. Fig 6 shows the 5-second return autocorrelation function on March 4-7, 2013 while Fig 7 shows the autocorrelation function of the returns on March 4, 2013 for four different sampling frequencies (i.e. upper left panel 5-second returns, upper right panel 10-second returns, lower left panel 30-second returns and lower right panel 1-minute returns). These two figures show a positive serial autocorrelation at high frequencies and this implies smaller estimates of the realized variance for these frequencies. As stressed in Ref. [25], high quality realized variance estimates can be constructed in a liquid market. However, we investigate the performance of the Fourier estimator also against illiquid assets.

**Fig 8:**
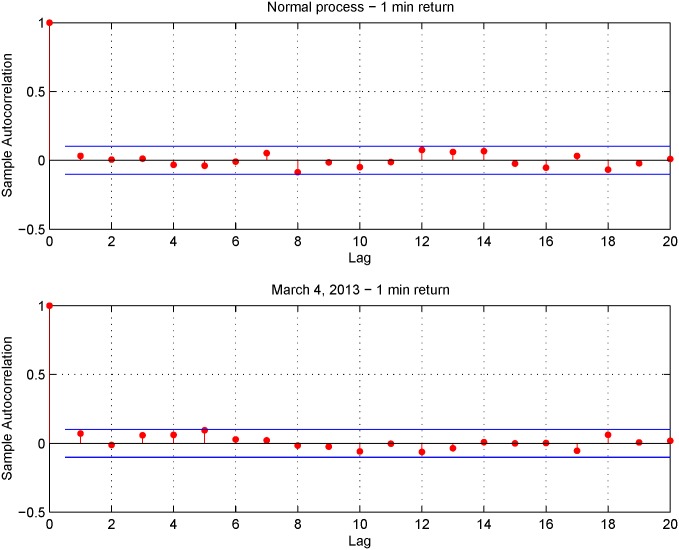
Comparison of the autocorrelation functions. Autocorrelation function of a sampled normal process Ne (0, Δ *t*) with sampling interval of 1 minute (upper panel) and of the 1-minute returns observed on March 4, 2013 (lower panel).

Fig 8 shows the autocorrelation function of a sampled Gaussian distribution N (0, Δ *t*) with sampling frequency of 1-minute (upper panel) and the autocorrelation of 1-minute observed returns on March 4, 2013 (lower panel). The results shown in the two panels and the preliminary analysis illustrated above suggest the use of 1-minute returns in order to reconstruct the distribution of the standardized return *z*_*t*_, defined in Eq (14). As previously mentioned, the standardized return, *z*_*t*_, is a standard Gaussian random variable when the prices are not affected by microstructure noise effects.

**Fig 9:**
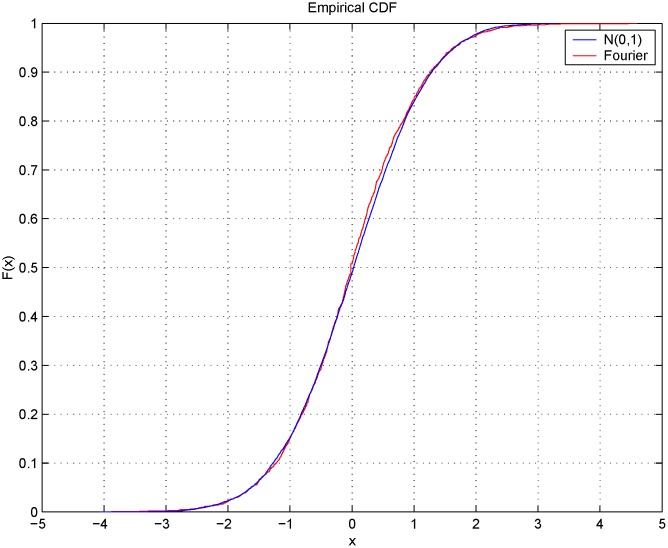
Comparison of the empirical cumulative distribution function and the standard normal one. Cumulative distribution functions of a sampled normal process N (0,1) (blue line) and of the sampled process $z_{t}^{\text{FR}}$ (red line) with a sampling interval of 1 minute (March 4-7, 2013).

We compute an estimate, $z_{t}^{\text{FR}}$, of the standardized return *z*_*t*_ using the spot volatility estimated with the Fourier method as done in Subsection 4.1. The frequencies *N* and *M* for the Fourier estimator are chosen according to *N* = *n*/2 and $M=\frac{1}{2\pi }\frac{1}{8}\sqrt{n}\text{log}\;n$. Fig 9 shows the empirical cumulative distribution function for $z_{t}^{\text{FR}}$ and the expected cumulative distribution function (i.e. N (0,1)), with Δ *t* = *T*/*n* = (1/390) seconds. Moreover, the BJ and the KS tests applied to the random sample $z_{t}^{\text{FR}}$ do not reject the null hypothesis at the significance level 0.05 and their p-values are 0.3, 0.6, respectively. The Fourier estimator shows a good performance in interpreting changes of volatilities despite the fact that the asset is illiquid. This finding is confirmed also by the second application, that is, the Value at Risk (VaR) prediction. This application has already been illustrated in Refs. [26, 28] to measure the performance of integrated volatility estimators and in Ref. [27] for the spot volatility estimators.

We apply the Fourier and TS estimators to predict 1%, 5%, 95%, 99% Value at Risk of 1-minute returns. As explained in Ref. [28], the conditional coverage probability must be the same as the theoretical level *α* of VaR in the left tail and 1 − *α* in the right tail. Hence, a measure of the performance is given by the difference between −*Q*(*α*) and *Q*(1 − *α*) where *Q*(⋅) is the quantile function of the empirical distribution of the standardized returns $z_{t}^{\text{FR}}$ and $z_{t}^{\text{TS}}$. Table 5 shows the conditional coverage probability (i.e. the empirical frequency that the failure of VaR prediction occurs) for the left and right tails of the 1-minute return distribution. Theoretically, we should expect a 1% and 5% rate of violation when we consider the 1% and 5% VaRs. We highlight the fact that the VaR predictions at 5% and 95% are satisfactory since we have two correct significant digits in the predictions.

**Table 5:** VaR prediction. Intraday VaR predictions obtained analyzing illiquid and liquid data with the Fourier and the TS estimators (sampling rate: one observation per minute).

	Illiquid Data		Liquid Data	
	Fourier	TS	Fourier	TS
1%	0.0119	0.0159	0.0087	0.0175
99%	0.0103	0.0144	0.0081	0.0148
5%	0.0484	0.0445	0.0508	0.0545
95%	0.0495	0.0520	0.0548	0.0572

### 5.2 Intraday variations of volatility when data are liquid

In this subsection we repeat the exercises of Subsection 5.1 using the nearby Italian stock index futures, FIB30, recorded every 5.67 seconds. Specifically, we consider four consecutive trading days (January 27-30, 2001). Quotes prior to 10 a.m. are removed to eliminate opening quotes from our sample.

**Fig 10:**
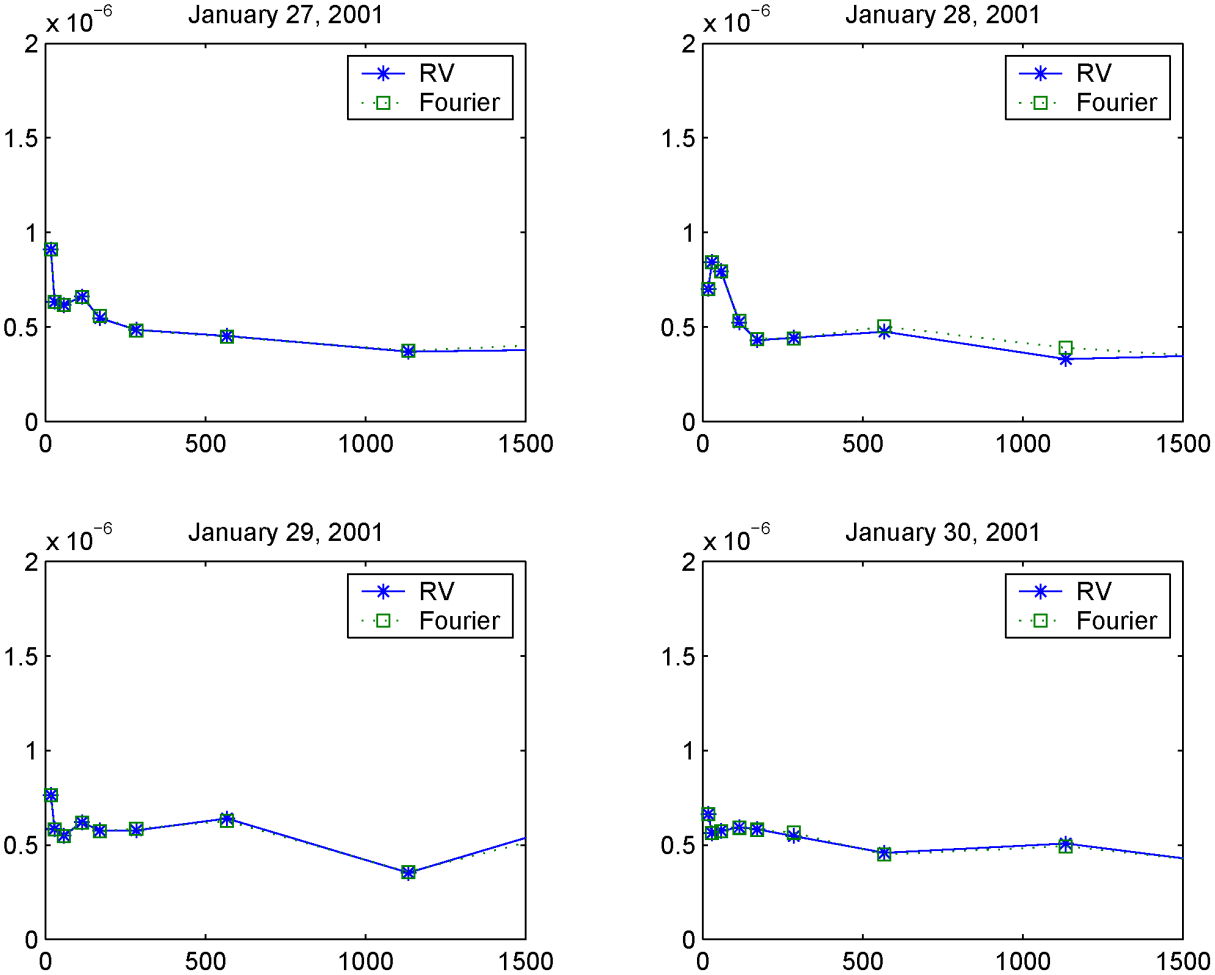
Volatility signature plots versus sampling frequency in seconds. The graphs show the signature plots of liquid market data on January 27-30, 2001

**Fig 11:**
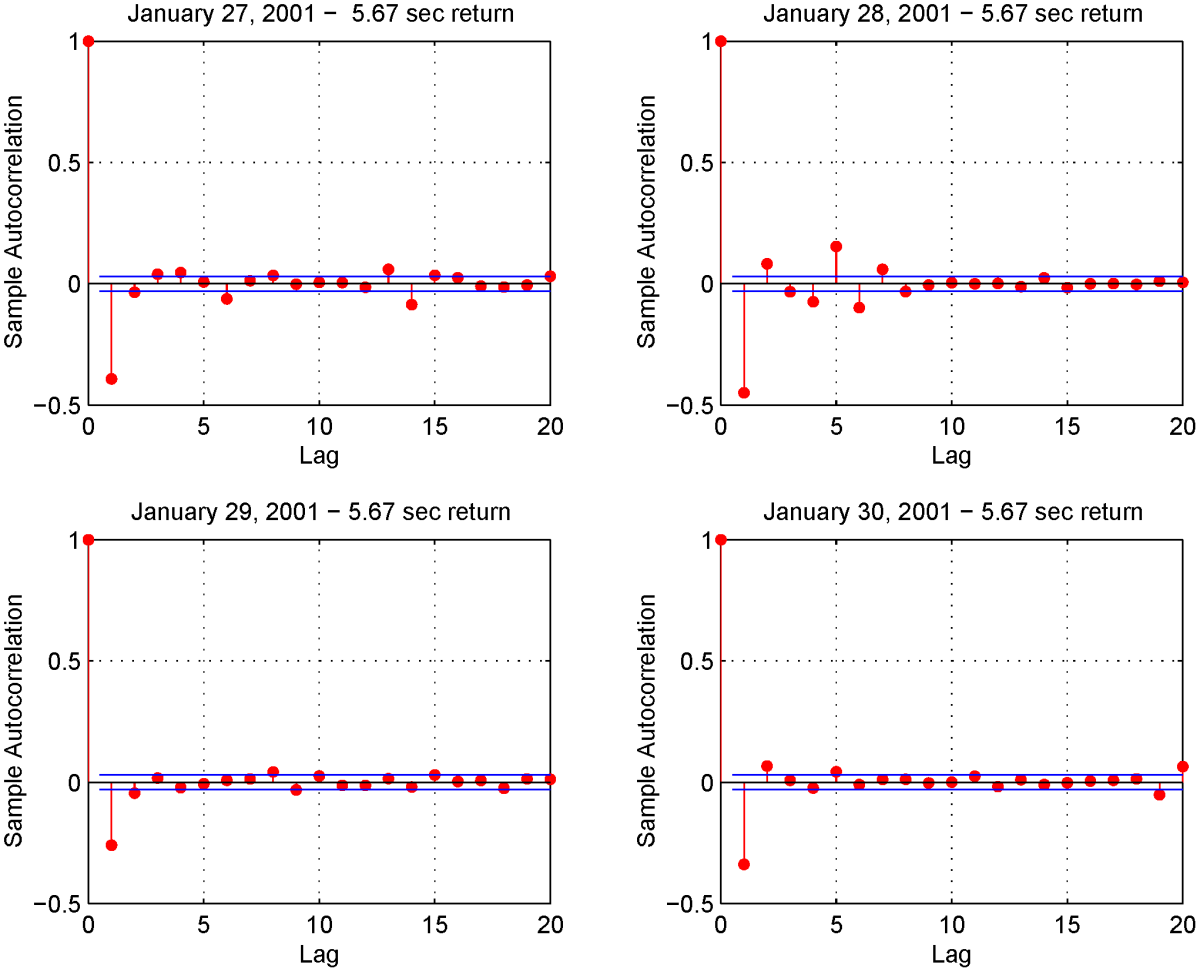
Autocorrelation functions. The four panels show the autocorrelation in 5.67-second returns on January 28-31, 2001.

**Fig 12:**
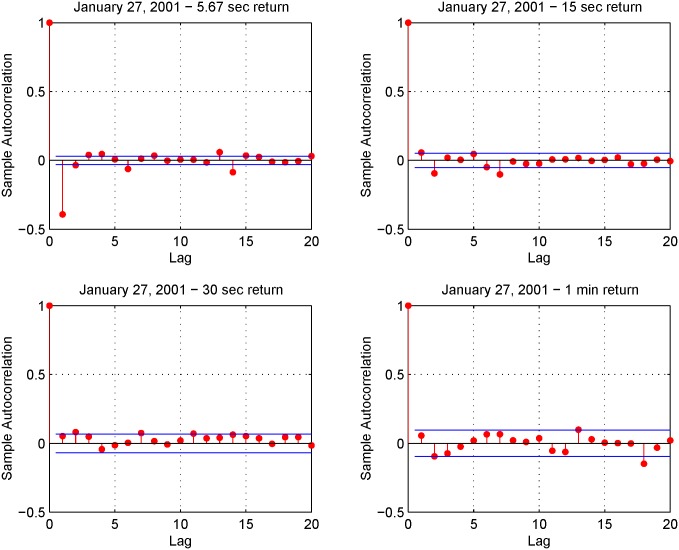
Autocorrelation for various sampling intervals on January 27, 2001. Upper left panel (5.67-second returns), upper right panel (15-second returns), lower left panel (30-second returns) and lower right panel (1-minute returns).

**Fig 13:**
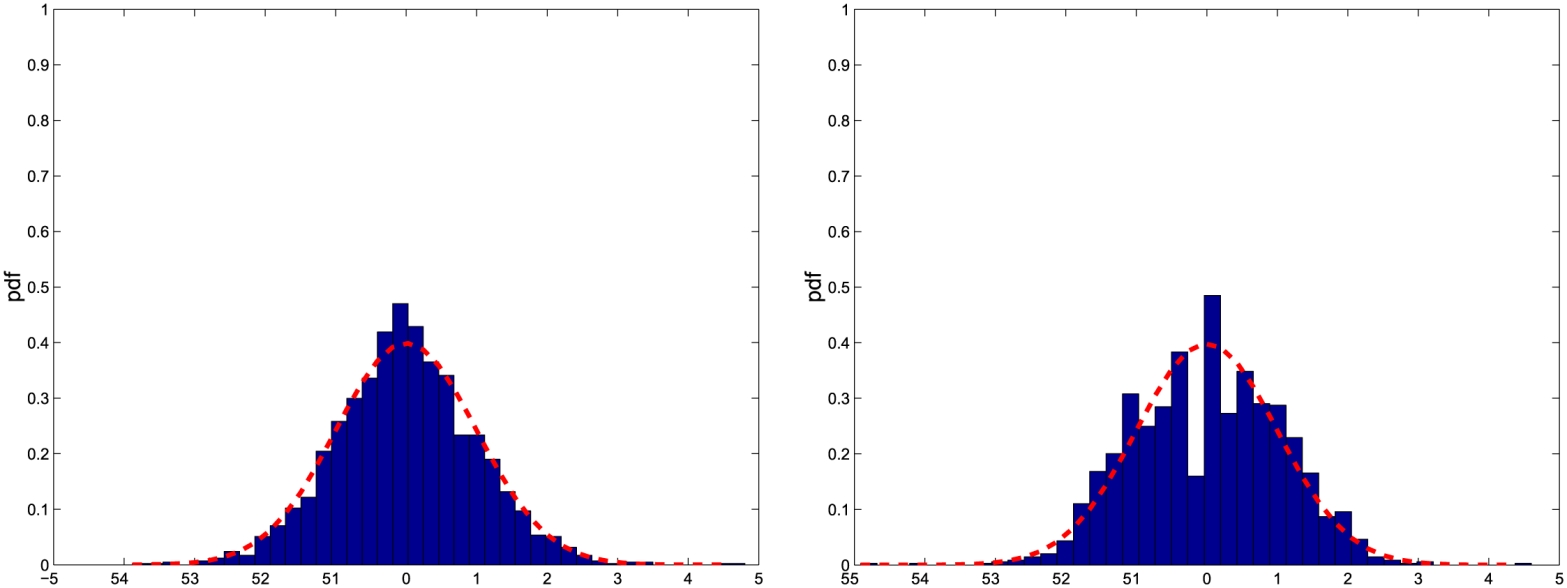
Empirical distribution functions. Left panel: pdf of a random variable N (0,1) (red dashed line) and of the sampled process $z_{t}^{\text{FR}}$ with a sampling interval of 1 minute (illiquid data). Right panel: pdf of a random variable N (0,1) (red dashed line) and of the sampled process $z_{t}^{\text{FR}}$ with a sampling interval of 1 minute (liquid data).

Fig 10 shows the volatility signature plots corresponding to January 27, 28, 29 and 30, 2001 evaluated using the Realized Variance (RV) and the Fourier estimator for the integrated volatility. The four plots in Fig 11 show the autocorrelation functions corresponding to the 5.67-second returns on January 27-30, 2001. We can see that the first-order autocorrelation is significantly negative while the second and third autocorrelations are slightly positive. The blue lines denote the 95% confidence interval. The shape of the signature plot of Fig 10 and the negative first order autocorrelation in Fig 11 show that FIB30 returns are liquid data. Fig 12 shows the autocorrelation function of the returns on January 27, 2001 for four different sampling frequencies (i.e. upper left panel 5.67-second returns, upper right panel 15-second returns, lower left panel 30-second returns and lower right panel 1-minute returns). As in Subsection 5.1, the preliminary analysis suggests the use of 1-minute returns to sample the standardized return, $z_{t}^{\text{FR}}$. Furthermore, the simple test illustrated in Section 4.2 selects the frequencies *N* = *n*/2 and $M=\frac{1}{2\pi }\frac{1}{8}\sqrt{n}\text{log}\;n$. Fig 13 shows the empirical distributions of the 1-minute standardized returns compared with the Gaussian density function obtained using illiquid data (Fig 13 left panel) and liquid data (Fig 13 right panel). The comparison of left and right panels highlights that the Fourier estimator provides accurate spot volatility estimates for both liquid and illiquid data. This is confirmed also by the VaR predictions shown in Table 5 (Liquid Data column), where the VaR predictions have at least two correct significant digits. Note that VaR predictions obtained using the TS estimators slightly outperform those obtained with Fourier estimator in the case of liquid data while the opposite happens in the case of illiquid data.

## 6 Conclusions

We have obtained the asymptotic error distribution of the Fourier spot volatility estimator with optimal rate of convergence and variance. Furthermore, extensive simulation studies and two empirical analysis have been proposed to show the efficiency of the Fourier estimator with high frequency data. The results illustrated in Sections 4 and 5 show that it is robust to various noise specifications. In addition, it is a *globally robust* estimator in the sense that it allows us to choose values of the so called cutting frequencies independently of the specific instant of time in the observed time window, of the specific features of the volatility process and of different noise models and levels. This feature of the Fourier estimator makes it particularly suited for empirical applications, such as the calibration of stochastic volatility models for asset prices, futures prices and models where the price volatility can provide insights into the traders’ strategies in the financial market. Finally, the high frequency intraday variations of volatility could be used to detect the onset of flash crashes (i.e. a deep fall in security prices occurring in a few seconds). The most relevant flash crash occurred on May 6th, 2010 when 4.1 billion dollars traded on the NYSE resulted in a drop of the Dow Jones Industrial Average of over 1000 points and then a rise to approximately the previous value. The mechanism which causes these events has been studied in depth and the high frequency spot volatility estimators could provide useful insights into this speculative trading.

## 7 Appendix A

**Remark 7.1** *Given the discrete time observations* {*t*_*j*_}_*j* = 0, …, *n*_, *denote* *ϕ*_*n*_(*τ*) := sup{*t*_*j*_ ∈ [0, *t*]:*t*_*j*_ ≤ *τ*}, *then using Itô formula we can write* Eq (6) *as*

$$\begin{array}{c} {\hat{\sigma }}_{n,N,M}^{2}\left(t\right)=\frac{1}{2\pi }\int_{0}^{2\pi }F_{M}\left(t-\phi_{n}\left(v\right)\right)\sigma^{2}\left(v\right)dv+F_{1}\left(t\right)+F_{2}\left(t\right), \end{array}$$ (24)

*where*

$$\begin{array}{c} F_{1}\left(t\right)=\frac{1}{2\pi }\int_{0}^{2\pi }F_{M}\left(t-\phi_{n}\left(v\right)\right)\int_{0}^{v}D_{N}\left(\phi_{n}\left(v\right)-\phi_{n}\left(u\right)\right)\sigma \left(u\right)dW_{u}\;\sigma \left(v\right)dW_{v} \end{array}$$ (25)

$$\begin{array}{c} F_{2}\left(t\right)=\frac{1}{2\pi }\int_{0}^{2\pi }\int_{0}^{v}F_{M}\left(t-\phi_{n}\left(u\right)\right)D_{N}\left(\phi_{n}\left(v\right)-\phi_{n}\left(u\right)\right)\sigma \left(u\right)dW_{u}\;\sigma \left(v\right)dW_{v}. \end{array}$$ (26)

*Proof.* (of Theorem 3.1) According to Lemma 2.2 in Ref. [15], we can assume that *b* = 0. Further, it is not restrictive to assume that the volatility process is a.s. bounded in [0,2*π*], then ess sup ‖*σ* ^2^‖_∞_ < ∞, where ‖*σ* ^2^‖_∞_ = sup_*t*_ *σ* ^2^(*t*).

Using the decomposition by Eqs (24), (25), (26), we write

$$\begin{array}{ccc} & & \sqrt{\frac{n}{M}}\left({\hat{\sigma }}_{n,N,M}^{2}\left(t\right)-\sigma^{2}\left(t\right)\right)=\sqrt{\frac{n}{M}}\;(\frac{1}{2\pi }\int_{0}^{2\pi }F_{M}\left(t-\phi_{n}\left(v\right)\right)\sigma^{2}\left(v\right)dv-\sigma^{2}\left(t\right))\\ & & \;+\sqrt{\frac{n}{M}}\;\left(F_{1}\left(t\right)+F_{2}\left(t\right)\right). \end{array}$$

The proof is divided into three steps.

**I)** We prove that

$$\begin{array}{c} \sqrt{\frac{n}{M}}\;(\frac{1}{2\pi }\int_{0}^{2\pi }F_{M}\left(t-\phi_{n}\left(v\right)\right)\sigma^{2}\left(v\right)dv-\sigma^{2}\left(t\right)) \end{array}$$ (27)

converges to 0 in probability. We write Eq (27) as

$$\begin{array}{c} \sqrt{\frac{n}{M}}\;(\frac{1}{2\pi }\int_{0}^{2\pi }F_{M}\left(t-\phi_{n}\left(v\right)\right)\sigma^{2}\left(v\right)dv-\frac{1}{2\pi }\int_{0}^{2\pi }F_{M}\left(t-v\right)\sigma^{2}\left(v\right)dv) \end{array}$$ (28)

$$\begin{array}{c} +\sqrt{\frac{n}{M}}\;(\frac{1}{2\pi }\int_{0}^{2\pi }F_{M}\left(t-v\right)\sigma^{2}\left(v\right)dv-\sigma^{2}\left(t\right)). \end{array}$$ (29)

For the term (28) it holds a.s.:

$$\begin{array}{c} \sqrt{\frac{n}{M}}\;|\frac{1}{2\pi }\int_{0}^{2\pi }F_{M}\left(t-\phi_{n}\left(v\right)\right)\sigma^{2}\left(v\right)dv-\frac{1}{2\pi }\int_{0}^{2\pi }F_{M}\left(t-v\right)\sigma^{2}\left(v\right)dv|\\ \leq \text{ess}\;\sup∥\sigma^{2}{∥}_{\infty }\sqrt{\frac{M}{n}}\to 0, \end{array}$$

by Lemma 8.1 i). Then, consider the *L* ^1^-norm of the term (29) and split it in two terms:

$$\begin{array}{ccc} & & \sqrt{\frac{n}{M}}\;E[\;|\frac{1}{2\pi }\int_{0}^{2\pi }F_{M}\left(t-v\right)\sigma^{2}\left(v\right)dv-\sigma^{2}\left(t\right)|\;]\\ & & \;\leq \sqrt{\frac{n}{M}}\;\frac{1}{2\pi }\int_{\left[0,2\pi \right]\cap \{|t-v|\leq \frac{2\pi }{M+1}\}}F_{M}\left(t-v\right)\;E[|\sigma^{2}\left(v\right)-\sigma^{2}\left(t\right)\left|\right]\;dv \end{array}$$ (30)

$$\begin{array}{c} +\sqrt{\frac{n}{M}}\;\frac{1}{2\pi }\int_{\left[0,2\pi \right]\cap \{|t-v|>\frac{2\pi }{M+1}\}}F_{M}\left(t-v\right)\;E[|\sigma^{2}\left(v\right)-\sigma^{2}\left(t\right)\left|\right]\;dv. \end{array}$$ (31)

Consider Eq (30). It is smaller than

$$\begin{array}{c} \sqrt{\frac{n}{M}}\;\frac{1}{2\pi }\int_{\left[0,2\pi \right]\cap \{|t-v|\leq \frac{2\pi }{M+1}\}}F_{M}\left(t-v\right)dv\;E[\underset{\left[0,2\pi \right]\cap \{|t-v|\leq \frac{2\pi }{M+1}\}}{\sup}\left|\sigma^{2}\left(v\right)-\sigma^{2}\left(t\right)\right|]\\ \leq \sqrt{\frac{n}{M}}\;\frac{1}{2\pi }\int_{\left[0,2\pi \right]\cap \{|t-v|\leq \frac{2\pi }{M+1}\}}F_{M}\left(t-v\right)dv\;\frac{C}{{\left(M+1\right)}^{\nu }}\leq C\sqrt{\frac{n}{M}}\frac{1}{M^{\nu }} \end{array}$$

which goes to zero as soon as 1 < *γ* < 1+2*ν* (this holds under the given assumptions). Note that the first inequality follows because the volatility path is *ν*-Hölder continuous. Consider Eq (31):

$$\begin{array}{c} \sqrt{\frac{n}{M}}\;\frac{1}{2\pi }\int_{\left[0,2\pi \right]\cap \{|t-v|>\frac{2\pi }{M+1}\}}F_{M}\left(t-v\right)\;\left|\sigma^{2}\left(v\right)-\sigma^{2}\left(t\right)\right|dv\\ \leq \sqrt{\frac{n}{M}}\;2\;\text{ess}\;\sup{∥\sigma^{2}∥}_{\infty }\;\frac{1}{2\pi }\int_{\left[0,2\pi \right]\cap \{|t-v|>\frac{2\pi }{M+1}\}}F_{M}\left(t-v\right)dv\leq C\;\sqrt{\frac{n}{M}}\;\frac{1}{M} \end{array}$$

where we have used Lemma 8.1 ii). This converges to zero as soon as 1 < *γ* < 3.

**II)** According to Ref. [45] we determine the variance of our asymptotic distribution, by studying

$$\begin{array}{c} \langle \sqrt{\frac{n}{M}}\;\left(F_{1}\left(t\right)+F_{2}\left(t\right)\right),\sqrt{\frac{n}{M}}\;\left(F_{1}\left(t\right)+F_{2}\left(t\right)\right)\rangle \end{array}$$

where 〈, 〉 denotes the quadratic covariation in [0, 2*π*]. This is composed of four terms, each of them leads to the same limit. We study the first one in detail, the remaining terms are similar. Consider

$$\begin{array}{ccc} & & \langle \sqrt{\frac{n}{M}}\;F_{1}\left(t\right),\sqrt{\frac{n}{M}}\;F_{1}\left(t\right)\rangle \\ & & \;=\frac{n}{M}\frac{1}{{\left(2\pi \right)}^{2}}\int_{0}^{2\pi }F_{M}^{2}\left(t-\phi_{n}\left(v_{2}\right)\right){\left(\int_{0}^{v_{2}}D_{N}\left(\phi_{n}\left(v_{2}\right)-\phi_{n}\left(v_{1}\right)\right)\sigma \left(v_{1}\right)dW_{v_{1}}\right)}^{2}\sigma^{2}\left(v_{2}\right)dv_{2}. \end{array}$$

Applying Itô formula, we get

$$\begin{array}{c} {\left(\int_{0}^{v_{2}}D_{N}\left(\phi_{n}\left(v_{2}\right)-\phi_{n}\left(v_{1}\right)\right)\sigma \left(v_{1}\right)dW_{v_{1}}\right)}^{2}=\int_{0}^{v_{2}}D_{N}^{2}\left(\phi_{n}\left(v_{2}\right)-\phi_{n}\left(v_{1}\right)\right)\sigma^{2}\left(v_{1}\right)dv_{1}\\ +2\int_{0}^{v_{2}}(\int_{0}^{v_{1}}D_{N}\left(\phi_{n}\left(v_{2}\right)-\phi_{n}\left(u\right)\right)\sigma \left(u\right)dW_{u})D_{N}\left(\phi_{n}\left(v_{2}\right)-\phi_{n}\left(v_{1}\right)\right)\sigma \left(v_{1}\right)dW_{v_{1}}. \end{array}$$

Let

$$\begin{array}{c} F_{11}\left(t\right):=\frac{1}{{\left(2\pi \right)}^{2}}\int_{0}^{2\pi }F_{M}^{2}\left(t-\phi_{n}\left(v_{2}\right)\right)\int_{0}^{v_{2}}D_{N}^{2}\left(\phi_{n}\left(v_{2}\right)-\phi_{n}\left(v_{1}\right)\right)\sigma^{2}\left(v_{1}\right)dv_{1}\;\sigma^{2}\left(v_{2}\right)dv_{2} \end{array}$$

$$\begin{array}{c} F_{12}\left(t\right):=\frac{1}{{\left(2\pi \right)}^{2}}\int_{0}^{2\pi }F_{M}^{2}\left(t-\phi_{n}\left(v_{2}\right)\right)\int_{0}^{v_{2}}(\int_{0}^{v_{1}}D_{N}\left(\phi_{n}\left(v_{2}\right)-\phi_{n}\left(u\right)\right)\sigma \left(u\right)dW_{u})\\ \times D_{N}\left(\phi_{n}\left(v_{2}\right)-\phi_{n}\left(v_{1}\right)\right)\sigma \left(v_{1}\right)dW_{v_{1}}\sigma^{2}\left(v_{2}\right)dv_{2}. \end{array}$$

We prove that, for any *t* fixed, in probability:

$$\begin{array}{c} \frac{n}{M}F_{11}\left(t\right)\to \frac{1}{3}\left(1+2\eta \left(c\right)\right)\sigma^{4}\left(t\right) \end{array}$$ (32)

where *η*(*c*) is equal to Eq (10), and

$$\begin{array}{c} \frac{n}{M}F_{12}\left(t\right)\to 0. \end{array}$$ (33)

We begin with Eq (32). Let $V:=2\pi \frac{1}{2}(1+2\eta (c))=\pi (1+\eta (c))$. We have:

$$\begin{array}{ccc} & & \left|\frac{n}{M}F_{11}\left(t\right)-V\;\frac{2}{3}\frac{1}{2\pi }\;\sigma^{4}\left(t\right)\right|\\ & & \;=|\frac{n}{M}\frac{1}{{\left(2\pi \right)}^{2}}\int_{0}^{2\pi }F_{M}^{2}\left(t-\phi_{n}\left(v_{2}\right)\right)\int_{0}^{v_{2}}D_{N}^{2}\left(\phi_{n}\left(v_{2}\right)-\phi_{n}\left(v_{1}\right)\right)\sigma^{2}\left(v_{1}\right)dv_{1}\;\sigma^{2}\left(v_{2}\right)dv_{2}-V\frac{2}{3}\;\frac{1}{2\pi }\sigma^{4}\left(t\right)|\\ & & \;\leq |\frac{1}{{\left(2\pi \right)}^{2}}\;\int_{0}^{2\pi }\frac{1}{M}F_{M}^{2}\left(t-\phi_{n}\left(v_{2}\right)\right)(n\int_{0}^{v_{2}}D_{N}^{2}\left(\phi_{n}\left(v_{2}\right)-\phi_{n}\left(v_{1}\right)\right)\sigma^{2}\left(v_{1}\right)dv_{1}-V\sigma^{2}\left(v_{2}\right))\sigma^{2}\left(v_{2}\right)dv_{2}| \end{array}$$ (34)

$$\begin{array}{c} +V|\frac{1}{{\left(2\pi \right)}^{2}}\int_{0}^{2\pi }\frac{1}{M}F_{M}^{2}\left(t-\phi_{n}\left(v_{2}\right)\right)\sigma^{4}\left(v_{2}\right)dv_{2}-\frac{2}{3}\frac{1}{2\pi }\sigma^{4}\left(t\right)|. \end{array}$$ (35)

Consider Eq (34): it is less than

$$\begin{array}{c} \frac{1}{{\left(2\pi \right)}^{2}}\int_{0}^{2\pi }\frac{1}{M}F_{M}^{2}\left(t-\phi_{n}\left(v_{2}\right)\right)\;|n\;\int_{0}^{v_{2}}D_{N}^{2}\left(\phi_{n}\left(v_{2}\right)-\phi_{n}\left(v_{1}\right)\right)\sigma^{2}\left(v_{1}\right)dv_{1}-V\sigma^{2}\left(v_{2}\right)|\;\sigma^{2}\left(v_{2}\right)dv_{2} \end{array}$$

which is *o*_*p*_(1) in virtue of Lemmas 8.1 iv) and 8.3.

Consider now Eq (35): by Lemma 8.1 iv), it holds in probability

$$\begin{array}{c} \lim_{n,M\to \infty }|\frac{1}{{\left(2\pi \right)}^{2}}\int_{0}^{2\pi }\frac{1}{M}F_{M}^{2}\left(t-\phi_{n}\left(v_{2}\right)\right)\sigma^{4}\left(v_{2}\right)dv_{2}-\frac{2}{3}\frac{1}{2\pi }\sigma^{4}\left(t\right)|=0. \end{array}$$

Finally, observe that $V\frac{2}{3}\frac{1}{2\pi }\sigma^{4}(t)=\frac{1}{3}(1+2\eta (c))\sigma^{4}(t)$.

We prove now Eq (33). Consider

$$\begin{array}{c} \frac{n}{M}\frac{1}{{\left(2\pi \right)}^{2}}\int_{0}^{2\pi }F_{M}^{2}\left(t-\phi_{n}\left(v_{2}\right)\right)\int_{0}^{v_{2}}(\int_{0}^{v_{1}}D_{N}\left(\phi_{n}\left(v_{2}\right)-\phi_{n}\left(u\right)\right)\sigma \left(u\right)dW_{u})\\ \times D_{N}\left(\phi_{n}\left(v_{2}\right)-\phi_{n}\left(v_{1}\right)\right)\sigma \left(v_{1}\right)dW_{v_{1}}\;\sigma^{2}\left(v_{2}\right)dv_{2}. \end{array}$$

Using Itô isometry, we have in *L* ^2^-norm:

$$\begin{array}{c} n^{2}E[{\left(\int_{0}^{v_{2}}(\;\int_{0}^{v_{1}}D_{N}\left(\phi_{n}\left(v_{2}\right)-\phi_{n}\left(u\right)\right)\sigma \left(u\right)dW_{u})D_{N}\left(\phi_{n}\left(v_{2}\right)-\phi_{n}\left(v_{1}\right)\right)\sigma \left(v_{1}\right)dW_{v_{1}}\right)}^{2}]\\ =n^{2}E[\int_{0}^{v_{2}}{\left(\;\int_{0}^{v_{1}}D_{N}\left(\phi_{n}\left(v_{2}\right)-\phi_{n}\left(u\right)\right)\sigma \left(u\right)dW_{u}\right)}^{2}D_{N}^{2}\left(\phi_{n}\left(v_{2}\right)-\phi_{n}\left(v_{1}\right)\right)\sigma^{2}\left(v_{1}\right)dv_{1}]\\ \leq ∥\sigma^{2}{∥}_{\infty }^{2}\;n^{2}\int_{0}^{v_{2}}\int_{0}^{v_{1}}D_{N}^{2}\left(\phi_{n}\left(v_{2}\right)-\phi_{n}\left(u\right)\right)du\;D_{N}^{2}\left(\phi_{n}\left(v_{2}\right)-\phi_{n}\left(v_{1}\right)\right)dv_{1}. \end{array}$$

Therefore, it is enough to observe that, as $\frac{N}{n}\to c$,

$$\begin{array}{c} n\int_{0}^{v_{2}}\;(n\int_{0}^{v_{1}}D_{N}^{2}\left(\phi_{n}\left(v_{2}\right)-\phi_{n}\left(u\right)\right)du)\;D_{N}^{2}\left(\phi_{n}\left(v_{2}\right)-\phi_{n}\left(v_{1}\right)\right)dv_{1}=o\left(1\right) \end{array}$$

combining Lemmas 8.3 ii) and iii). Finally, remark that $\frac{1}{M}\int_{0}^{2\pi }F_{M}^{2}(t-\phi_{n}(v_{2}))dv_{2}=O(1)$, by Lemma 8.1 iii). This concludes the proof.

**III)** The last step of the proof requires to prove the convergence in probability

$$\begin{array}{c} \langle \sqrt{\frac{n}{M}}\;\left(F_{1}\left(t\right)+F_{2}\left(t\right)\right),W\rangle \to 0. \end{array}$$

Observe that:

$$\begin{array}{c} \langle \sqrt{\frac{n}{M}}\;F_{1}\left(t\right),W\rangle =\\ \;\;\;\;\;\sqrt{\frac{n}{M}}\frac{1}{2\pi }\int_{0}^{2\pi }F_{M}\left(t-\phi_{n}\left(v_{2}\right)\right)\int_{0}^{v_{2}}D_{N}\left(\phi_{n}\left(v_{2}\right)-\phi_{n}\left(v_{1}\right)\right)\sigma \left(v_{1}\right)dW_{v_{1}}\sigma \left(v_{2}\right)dv_{2}. \end{array}$$

Consider the *L* ^1^-norm:

$$\begin{array}{ccc} & & E[|\frac{1}{2\pi }\int_{0}^{2\pi }F_{M}\left(t-\phi_{n}\left(v_{2}\right)\right)\int_{0}^{v_{2}}D_{N}\left(\phi_{n}\left(v_{2}\right)-\phi_{n}\left(v_{1}\right)\right)\sigma \left(v_{1}\right)dW_{v_{1}}\sigma \left(v_{2}\right)dv_{2}|]\\ & & \;\leq {∥\sigma ∥}_{\infty }\frac{1}{2\pi }\int_{0}^{2\pi }F_{M}\left(t-\phi_{n}\left(v_{2}\right)\right)E[|\int_{0}^{v_{2}}D_{N}\left(\phi_{n}\left(v_{2}\right)-\phi_{n}\left(v_{1}\right)\right)\sigma \left(v_{1}\right)dW_{v_{1}}|]dv_{2}. \end{array}$$ (36)

Moreover, we have

$$\begin{array}{c} E[|\int_{0}^{v_{2}}D_{N}\left(\phi_{n}\left(v_{2}\right)-\phi_{n}\left(v_{1}\right)\right)\sigma \left(v_{1}\right)dW_{v_{1}}|]\leq {∥\sigma^{2}∥}_{\infty }^{\frac{1}{2}}{\left(\int_{0}^{v_{2}}D_{N}^{2}\left(\phi_{n}\left(v_{2}\right)-\phi_{n}\left(v_{1}\right)\right)dv_{1}\right)}^{\frac{1}{2}}. \end{array}$$

Thus

$$\begin{array}{ccc} & & \sqrt{\frac{n}{M}}E[|\frac{1}{2\pi }\int_{0}^{2\pi }F_{M}\left(t-\phi_{n}\left(v_{2}\right)\right)\int_{0}^{v_{2}}D_{N}\left(\phi_{n}\left(v_{2}\right)-\phi_{n}\left(v_{1}\right)\right)\sigma \left(v_{1}\right)dW_{v_{1}}\sigma \left(v_{2}\right)dv_{2}|]\\ & & \;\leq C\sqrt{\frac{1}{M}}\int_{0}^{2\pi }F_{M}\left(t-\phi_{n}\left(v_{2}\right)\right){\left(n\int_{0}^{v_{2}}D_{N}^{2}\left(\phi_{n}\left(v_{2}\right)-\phi_{n}\left(v_{1}\right)\right)dv_{1}\right)}^{\frac{1}{2}}. \end{array}$$ (37)

Using Lemma 8.3 i)

$$\begin{array}{c} \lim_{n,N\to \infty }n\int_{0}^{v_{2}}D_{N}^{2}\left(\phi_{n}\left(v_{2}\right)-\phi_{n}\left(v_{1}\right)\right)dv_{1}\leq C_{2} \end{array}$$

therefore, Eq (37) is less than

$$\begin{array}{c} C\sqrt{\frac{1}{M}}\int_{0}^{2\pi }F_{M}\left(t-v_{2}\right)dv_{2}\leq C\sqrt{\frac{1}{M}}\;2\pi \to 0\;\;as\;M\to \infty . \end{array}$$

□

## 8 Appendix B

This Appendix contains some results about the Fejer and Dirichlet kernels: these results are known but we place them here for the reader’s convenience.

**Lemma 8.1** (*Fejer kernel properties*)

- *For any* *M*
  $$\begin{array}{c} \int_{-\pi }^{\pi }F_{M}\left(x\right)dx=2\pi . \end{array}$$
  *Moreover, under the assumption* ${\text{lim}}_{n,M\to \infty }\frac{M^{\gamma }}{n}=a>0$ *for some* *γ* > 1, *then*
  $$\begin{array}{c} \lim_{n,M\to \infty }\int_{-\pi }^{\pi }F_{M}\left(\phi_{n}\left(x\right)\right)dx=2\pi , \end{array}$$
  *in particular*,
  $$\begin{array}{c} |\int_{-\pi }^{\pi }F_{M}\left(\phi_{n}\left(x\right)\right)dx-\int_{-\pi }^{\pi }F_{M}\left(x\right)dx|\leq C\frac{M}{n}. \end{array}$$
- *For any* *M* ≥ 1
  $$\begin{array}{c} \int_{\frac{2\pi }{M+1}}^{\pi }F_{M}\left(x\right)dx\leq \frac{C}{M}. \end{array}$$
- *If* ${\text{lim}}_{n,M\to \infty }\frac{M^{\gamma }}{n}=a>0$ *for some* *γ* > 1
  $$\begin{array}{c} \lim_{M,n\to \infty }\int_{-\pi }^{\pi }\frac{1}{M}F_{M}^{2}\left(\phi_{n}\left(x\right)\right)dx=\lim_{M\to \infty }\int_{-\pi }^{\pi }\frac{1}{M}F_{M}^{2}\left(x\right)dx=\frac{4\pi }{3}. \end{array}$$ (38)
- *If* ${\text{lim}}_{n,M\to \infty }\frac{M^{\gamma }}{n}=a>0$ *for some* *γ* > 1 *and* *σ* ^2^ *is Holder continuous with parameter* *ν* ∈ (0,1], *then*
  $$\begin{array}{c} \lim_{n,M\to \infty }\frac{1}{M}\int_{-\pi }^{\pi }F_{M}^{2}\left(t-\phi_{n}\left(s\right)\right)\sigma^{2}\left(s\right)ds=\lim_{M\to \infty }\int_{-\pi }^{\pi }\frac{1}{M}F_{M}^{2}\left(t-s\right)\sigma^{2}\left(s\right)ds=\frac{4\pi }{3}\sigma^{2}\left(t\right). \end{array}$$ (39)

*Proof.* The proofs can be found e.g. in Ref. [23], in order (5.9), Lemma 6.1, (5.10) or Remark 5.2, Lemma 5.1. □

**Remark 8.2** *As in Ref*. [23] *Remark 5.2 all above results are true in* [0, *T*] *very similarly*.

**Lemma 8.3**

- *If* ${\text{lim}}_{n,N\to \infty }\frac{N}{n}=c\neq 0$, *then for any* *p* > 1 *there exists a constant* *C*_*p*_ *such that*
  $$\begin{array}{c} \lim_{n,N}\;n\underset{v_{2}\in \left[0,2\pi \right]}{\sup}\int_{0}^{2\pi }D_{N}^{p}\left(\phi_{n}\left(v_{2}\right)-\phi_{n}\left(v_{1}\right)\right)dv_{1}\leq C_{p}. \end{array}$$
- *If* ${\text{lim}}_{n,N\to \infty }\frac{N}{n}=c\neq 0$, *then*
  $$\begin{array}{c} \lim_{n,N\to \infty }n\;\int_{0}^{v_{2}}D_{N}^{2}\left(\phi_{n}\left(v_{2}\right)-\phi_{n}\left(v_{1}\right)\right)dv_{1}=\pi \left(1+\eta \left(c\right)\right). \end{array}$$
- *If* ${\text{lim}}_{n,N\to \infty }\frac{N}{n}=c\neq 0$, *then for any* *v*_1_ < *v*_2_ *we have*
  $$\begin{array}{c} \lim_{N,n}\;n\int_{0}^{v_{1}}D_{N}^{2}\left(\phi_{n}\left(v_{2}\right)-\phi_{n}\left(u\right)\right)du=0. \end{array}$$
  *Moreover, if* *σ* ^2^ *is Holder continuous with parameter* *ν* ∈ (0,1], *also holds for any* *v*_1_ < *v*_2_
  $$\begin{array}{c} \lim_{n,N\to \infty }\;n\int_{0}^{v_{1}}D_{N}^{2}\left(\phi_{n}\left(v_{2}\right)-\phi_{n}\left(u\right)\right)\sigma^{2}\left(u\right)du=0. \end{array}$$

*Proof.* The proofs can be found e.g. in Ref. [21] Lemma 3, Lemma 1 and Lemma 4(1), in order. □
